# Novel D-form of hybrid peptide (D-AP19) rapidly kills *Acinetobacter baumannii* while tolerating proteolytic enzymes

**DOI:** 10.1038/s41598-022-20236-1

**Published:** 2022-09-23

**Authors:** Phanvimon Jariyarattanarach, Natthaporn Klubthawee, Mathira Wongchai, Sittiruk Roytrakul, Ratchaneewan Aunpad

**Affiliations:** 1 Graduate Program in Biomedical Sciences, Faculty of Allied Health Sciences, Thammasat University, Khlong Luang, Pathum Thani 12120 Thailand; 2 Functional Ingredients and Food Innovation Research Group, National Center for Genetic Engineering and Biotechnology, National Science and Technology Development Agency, Khlong Luang, Pathum Thani 12120 Thailand

**Keywords:** Antibiotics, Applied microbiology

## Abstract

Antimicrobial peptides (AMPs) are being developed as potent alternative treatments to conventional antibiotics which are unlikely to induce bacterial resistance. They can be designed and modified to possess several druggable properties. We report herein a novel hybrid peptide of modified aurein (A3) and cathelicidin (P7), or A3P7, by a flipping technique. It exhibited potent antibacterial activity against both Gram-negative and -positive pathogenic bacteria but had moderate hemolytic activity. To reduce the sequence length and toxicity, C-terminal truncation was serially performed and eight truncated derivatives (AP12–AP19) were obtained. They had significantly less hemolytic activity while preserving antibacterial activity. Secondary structures of the candidate peptides in environments simulating bacterial membranes (30 mM SDS and 50% TFE), determined by CD spectroscopy, showed α-helical structures consistent with predicted in silico 3D structural models. Among the peptides, AP19 demonstrated the best combination of broad-spectrum antibacterial activity (including toward *Acinetobacter baumannii*) and minimal hemolytic and cytotoxic activities. A D-form peptide (D-AP19), in which all L-enantiomers were substituted with the D-enantiomers, maintained antibacterial activity in the presence of pepsin, trypsin, proteinase K and human plasma. Both isomers exhibited potent antibacterial activity against multi-drug (MDR) and extensively-drug resistant (XDR) clinical isolates of *A. baumannii* comparable to the traditional antibiotic, meropenem. D-AP19 displayed rapid killing via membrane disruption and leakage of intracellular contents. Additionally, it showed a low tendency to induce bacterial resistance. Our work suggested that D-AP19 could be further optimized and developed as a novel compound potentially for fighting against MDR or XDR *A. baumannii*.

## Introduction

The growing global crisis of antimicrobial-resistant (AMR) bacteria is causing enormous harm to human health, especially in prolonging and complicating the hospital stays of surgical patients^1^. Hospital-acquired infections (HAIs) with these organisms occur in several ways. For example, exposure with an infected person, a contaminated environment, or a non-sterile invasive device (such as catheters and ventilators)^2^. These widespread HAIs cause more morbidity and mortality, economic loss, and extensive complications of patient status and treatment^2^. Research and development of novel antimicrobial agents to fight against drug-resistant bacteria was declared as urgently needed for the global health care system by the World Health Organization (WHO), targeting *Acinetobacter baumannii* as the first priority^3^. During the Covid-19 (coronavirus disease-2019) pandemic era, excessive use of antibiotics has caused an increase in the incidence of AMR bacteria, including *A. baumannii*^4^. Worryingly, bacterial infections can co-infect patients with Covid-19 and increase morbidity–mortality rates^5^. Thus, the development of novel antimicrobial agents for treatment of multi-drug resistant bacteria is crucial.

Cationic antimicrobial peptides (CAMPs) are naturally occurring peptides, and crucial components of the innate immune system^6^. They contribute to rapid clearance of biological agents through direct killing of the organisms, inhibition of pro-inflammatory mediators such as lipopolysaccharide, and by modulating inflammatory responses to infection^6^. These multi-targeted actions can prevent the occurrence of resistant bacteria, unlike conventional antibiotics that have specific targets and so higher likelihoods of inducing bacterial resistance^7^. The ability of CAMPs to control infections and resolve harmful inflammation make them attractive for development as alternative strategies to combat drug-resistant pathogens^8^. Among their structural classes, α-helical peptides with cationic and amphipathic properties exhibit broad-spectrum antibacterial activity against both Gram-negative and -positive bacteria^9^. However, they may also have unfavorable properties such as high toxicity or low stability in physiological conditions. Due to their uncomplicated composition of amino acids linked with peptide bonds, AMPs can be easily designed or modified to possess properties such as high antimicrobial activity, low toxicity to mammalian cells, and high stability to proteolysis^10^.

The major parameters influencing the activity of AMPs are total net charge, hydrophobicity, amphipathicity and helicity, which can damage cell membrane structures and result in cell lysis or pore formation^8,10^. Changing one parameter may affect another, so changes need to be made and optimized carefully. The modification of a peptide sequence generally alters more than one structural characteristic that might modulate antimicrobial activity. As different amino acids have different physiological characteristics, an amino acid substitution at a key position can improve the potency/selectivity balance^11^. C-terminal truncation is a strategy to cleave amino acids from the C terminus of the peptide sequence to improve antimicrobial activity and reduce production cost^12^. In particular, some modifications are exploited for a specific objective, such as D-amino acid substitution. This is the incorporation of D-amino acids (non-natural form of an amino acid) into AMP sequences, which will reverse the stereochemistry of the peptide (or adopt a right-handed α-helical configuration) and hence prevent protease degradation and promote peptide stability^13^. In addition, the replacement of D-amino acids tends to alter structure and amphipathicity without effecting hydrophobicity and net charge^14^. The partial D-amino acid substitution of the W3R6 peptide enhances tolerance to proteases without altering its antimicrobial activity^15^. D-amino acid substitution of the mammalian HBcARD peptide improved its stability and antimicrobial activity while maintaining very low hemolytic effect^16^.

The parent α-helical peptides P0 and A0 were designed from the conserved sequences of 45 α-helical cathelicidin and 11 α-helical aurein, respectively, by a template-assisted approach^21^. They were further modified to P7 and A3 derivatives by truncation and amino acid substitution with hydrophobic and positively charged amino acids, notably tryptophan and arginine. The peptide analogues of aurein 2.2, rich in arginine and tryptophan, were designed and found to be more active than the parent peptide^17^. A hybrid peptide, modified from α-helical cathelicidin (P7) and aurein (A3) and designated as P7A3, showed promising antimicrobial activity with potential for application^18^. In this study, the hybrid peptide analogue, A3P7, was obtained by a flipping technique and serially C-terminal truncated to obtain an optimal peptide candidate, AP19. Stability toward proteolytic enzymes was enhanced by D-amino acid substitution (D-AP19). The novel peptide rapidly killed *A. baumannii* ATCC 19,606 via membrane disruption and had a low tendency to induce bacterial resistance. It also exhibited potent antibacterial activity against multidrug-resistant (MDR) and extensively drug-resistant (XDR) clinical isolates of *A. baumannii*.

## Materials and methods

### Peptide design and sequence analysis

In our previous study, the hybrid P7A3 was designed by hybridizing modified cathelicidin, P7, with modified aurein, A3, at the C-terminus^18^. Since P7 showed much higher antimicrobial activity than A3, we opted to maintain the sequence of P7 and augment its activity by hybridizing with A3 at the C-terminus. However, the reverse conjugation (by hybridizing A3 with P7 at the C-terminus and obtaining A3P7) also showed promising antibacterial activity, comparable to that of P7A3. This reverse conjugation approach was designated as ‘flipping technique’ in the present work. Amino acid sequence analysis was conducted using the programs ProtParam (ExPASy Proteomics Server: https://web.expasy.org/protparam/) and Antimicrobial Peptide Calculator and Predictor (APD3 Server: http://aps.unmc.edu/AP/prediction/prediction_main.php). Its three-dimensional structure was predicted by I-TASSER (http://zhanglab.ccmb.med.umich.edu/I-TASSER/). The helical wheel projection was calculated using the online program NetWheels: Peptides Helical Wheel and Net Projections Maker (http://lbqp.unb.br/NetWheels/). The D-forms of AP19 or D-AP10 were obtained by substituting all L-amino acids with D-amino acids.

### Peptide synthesis and preparation

The peptides used in this study were synthesized by a solid-phase method using 9-fluorenylmethoxycarbonyl (Fmoc) chemistry and purified by HPLC as trifluoroacetate salts with purities > 95% (ChinaPeptides, China). Purity and mass were verified by analytical reversed-phase HPLC and electrospray ionization mass spectrometry (ESI–MS), respectively. The peptide was dissolved in deionized water and stored as a stock at concentration of 10 mg/ml. Peptides were stored at − 20 °C until subsequent experiments and dilutions were freshly prepared.

### Bacterial strains and growth conditions

Ten strains of both Gram-positive and -negative bacteria, including *Staphylococcus aureus* ATCC 25,923, *S. epidermidis* ATCC 12,228, *Bacillus cereus* ATCC 11,778, *Listeria monocytogenes* 10403S, *Salmonella typhimurium* ATCC 13,311, *Pseudomonas aeruginosa* ATCC 27,853, *Shigella sonnei* ATCC 11,060, *A. baumannii* ATCC 19,606, *Escherichia coli* ATCC 25,922 and *E. coli* O157:H7, were used in this study. Eight clinical isolates of multidrug-resistant (MDR) and extensively drug-resistant (XDR) *A. baumannii,* isolated from human (blood, sputum, respiratory sample, sterile site sample and urine), were kindly provided by Asst. Prof. Dr. Sakawrat Kanthawong (Faculty of Medicine, Khon Kaen University, Thailand) (Table S1). All bacterial strains, excluding *L. monocytogenes* 10403S and *E. coli,* were cultured in tryptic soy broth (TSB). *L. monocytogenes* was grown in TSB supplemented with 0.6% yeast extract (TSB-YE), and *E. coli* was cultured in Luria broth (LB). All of bacteria were cultured at 37 °C with continuous shaking at 220 rpm.

### Antimicrobial assays

Minimum inhibitory concentration (MIC) of peptides and antibiotics was assessed by a modified version of the broth microdilution method from the Clinical and Laboratory Standards Institute (CLSI) as previously described^19^. In brief, mid-log phase growths of microbial strains were cultured in Müeller-Hinton broth (MHB) with an initial inoculum of 2–8 × 10^5^ CFU/mL. Bacteria suspensions were incubated in sterile 96-well plates with test agents at desired concentrations. MIC was defined as the lowest concentration of peptide that prevented visible growth of bacteria after 24 h incubation with continuous shaking (220 rpm) at 37 °C. Minimum bactericidal concentration (MBC) determination was performed using a modified version of the colony count assay^19^. In brief, fifty µl of all non-turbid wells from MIC experiments were spread on agar plates. The MBC value was defined as the lowest concentration of peptides with no colony growth on the plate. All assays were performed in triplicate.

### Hemolysis assay

The hemolytic activity of peptides was determined as the amount of hemoglobin released from lysed human red blood cells (hRBCs) after treatment with peptide^20^. Fresh hRBCs were collected from a healthy volunteer in polycarbonate tubes containing heparin. Then, the collected packed RBCs were washed at least three times (or until the supernatant was clear) with sterile phosphate-buffered saline pH 7.4 (PBS) and centrifuged at 2,000 × g for 5 min. The 2% (v/v) of washed hRBCs in PBS were incubated with serially diluted peptides (0.98 to 250 μg/ml) for 1 h at 37 °C. After centrifugation, the supernatants were monitored for optical density (OD) at 405 nm using a Multiskan FC Microplate Reader^21^. Values for 0% (negative control) and 100% (positive control) lysis were determined by incubating the hRBCs with PBS only (OD_Blank_) and 0.1% (v/v) Triton X-100 (OD_Triton X-100_), respectively. The experiments were performed in triplicate. The percent hemolysis was calculated according to the following equation:

$$ \% {\text{Hemolysis}} = \left( {{\text{OD}}_{{{\text{Sample}}}} {-}{\text{ OD}}_{{{\text{Blank}}}} } \right)/\left( {{\text{OD}}_{{{\text{Triton X}} - {1}00}} {-}{\text{OD}}_{{{\text{Blank}}}} } \right) \times {1}00 $$

Experiments associated with human volunteers were carried out in accord with the ethical standards of and approved by the Ethics Committee of Thammasat University (COA No. 066/2562). The informed consent was voluntarily written by all individual participants.

### In vitro cytotoxicity assay

The colorimetric 3-(4,5 dimethylthiazol-2-yl)-2,5-diphenyltetrazolium bromide (MTT; Invitrogen) dye reduction assay was used to determine the cytotoxicity of peptides on mouse fibroblast (L929) cells (NCTC clone 929) according to a modified MTT assay^22^. L929 cells were cultured in Dulbecco’s Modified Eagle Medium (DMEM; Gibco) containing 10% fetal bovine serum (FBS; Gibco) and 100 U/ml penicillin–streptomycin in a fully humidified atmosphere of 95% air and 5% CO_2_ at 37 °C. The cells (10^5^ cells/well) were seeded on 96-well plates and incubated with serially diluted peptide (0.98 to 250 μg/ml) for 24 h. At the end of incubation period, MTT solution (100 µl, 0.4 mg/ml) was added to each well and incubated for 4 h. The supernatants were removed and replaced by 100 µl of DMSO to dissolve the purple formazan crystals. Absorbance was measured using a MultiskanTM FC microplate reader at a wavelength of 570 nm. Cells without peptides served as negative controls. Cell viability (percentage) was calculated using the following equation:

$$ \% {\text{ Cell viability}} = \left( {{\text{OD}}_{{{57}0\,{\text{of treated sample}}}} - {\text{OD}}_{{{\text{Blank}}}} } \right)/\left( {{\text{OD}}_{{{57}0\,\,{\text{of control}}}} - {\text{OD}}_{{{\text{Blank}}}} } \right) \times {1}00\% $$

### Assessment of peptide stability in different environments

The MIC and MBC assays were performed after peptide was exposed to various conditions, including proteolytic enzymes, serum salts and human plasma^23^. AP19 and D-AP19 at final concentration ranging from 0.98 to 250 µg/ml were pre-incubated with a 1 mg/ml final concentration of proteolytic enzyme (trypsin, pepsin and proteinase K) at 37 °C for 1 h. To determine peptide sensitivity to physiological salts, approximately 5 × 10^5^ CFU/ml of *A. baumannii* ATCC 19,606 was mixed with the MHB containing different physiological salts at final concentration as follows: 150 mM NaCl, 4.5 mM KCl, 1 mM MgCl_2_, 6 µM NH_4_Cl, 8 µM ZnCl_2_, 4 µM FeCl_3_ and 2.5 µM CaCl_2_. After treatment, the MIC was evaluated as described in 2.4. To test the stability of peptides in human plasma, peptide was mixed with pure human plasma, and the mixture was incubated for 1 h at 37 °C. Then, the mixture was two-fold serially diluted with sterile MHB. The peptides at final concentrations of 0.98 to 250 µg/ml were incubated with an equal volume of bacterial suspension at 37 °C for 24 h. MIC values were determined as described in 2.4.

### Time-kill kinetic assays

The kinetics of the bactericidal activity of D-AP19 against *A. baumannii* ATCC 19,606 were determined by assessment of the time course of bacterial killing. A bacterial inoculum at approximately 5 × 10^5^ CFU/ml suspended in MHB was incubated with D-AP19 at a concentration of 7.81 µg/ml (both MIC and MBC) at 37 °C with continuous shaking at 220 rpm. Samples were taken from the 96-well plates at specific time intervals (0, 0.25, 0.5, 1, 2, 4, 6, 8, 10, 12 and 24 h) and tenfold serially diluted in PBS. Aliquots of each dilution were then plated on tryptic soy agar (TSA). Colonies of bacteria were counted after overnight incubation. A control of bacterial growth (no peptide added) was included in each run.

### Circular dichroism analysis

The secondary structure of selected designed peptides dissolved in PBS was determined using circular dichroism (CD) spectra on a Jasco-815 spectropolarimeter under nitrogen at 25 °C, using a 0.1-cm-path-length rectangular quartz cell^24^. Peptide spectra were recorded in 3 different environments: PBS, 30 mM sodium dodecyl sulfate (SDS) micelles in PBS, and 50% (v/v) TFE (2,2,2-trifluoroethanol) in PBS. The SDS micelles simulate the anionic amphipathic environment of bacterial membranes, i.e., external negatively charged surface, but hydrophobic internal environment like the chains of phospholipids. The fluorinated environment poorly interacts with the molecule, and therefore promotes intra-molecular interactions, that often end in a forced structuration (e.g., in alpha-helix) of the peptide. Spectra were determined in triplicate using a 190–260 nm range at a scanning speed of 10 nm/min. After that, the acquired CD signal spectra were converted to mean residue ellipticity using the following equation:

$$ \theta_{M} = \, (\theta_{obs} /{1}0) \, \times \, ({\text{M}}_{RW} /c \cdot {1}) $$

where θ_M_ is residue ellipticity (deg. M-1 m-1), θ_obs_ is detected ellipticity adjusted for buffer at a given wavelength (mdeg), M_RW_ is residue molecular weight (M_W_/number of amino acids), c is peptide concentration (mg/mL), and l is path length (cm).

In addition, the CD spectra were analyzed by CDPro software to estimate the content of secondary structures. The CONTIN/LL from the software package was used to analyze the data.

### Flow cytometry analysis

Damage to bacterial cell membranes after interaction with peptide was evaluated using flow cytometry with incorporation of fluorescent dyes^25^. Mid-log phase growth of *A. baumannii* ATCC 19,606 at OD_620_ 0.05 in MHB was treated with 0.5 × MIC and 1 × MIC of D-AP19, followed by incubation at 37 °C for 0 h, 0.25 h, 1 h or 2 h with continuous shaking at 220 rpm. The treated bacterial cells were centrifuged at 10,000 × g for 10 min. Then, they were washed again to remove unbound-peptide molecules. Each sample was stained with PI (propidium iodide) or BOX (bis-(1,3-dibutylbarbituric acid) trimethine oxonol) fluorescent dye. All data were recorded using a flow cytometer (CytoFlex, Beckman Coulter), counting 25,000 cells in each sample, at a laser excitation wavelength of 488 nm. Forward scatter (FS) and side scatter (SS) indicated cell size and granularity (complexity), respectively. Red (585/342 nm) and green (530/30 nm) fluorescent signals from PI and BOX, respectively, were investigated. The data were analyzed with Kaluza software version 2.1 (Beckman Coulter, Brea, CA, United States). Negative and positive controls were untreated bacterial cells at 0 h and bacterial cells heated for 30 min at 70 °C, respectively. Three independent experiments were performed.

### Scanning electron microscopy

Morphologic changes of bacterial cell surfaces after D-AP19 treatment were investigated by scanning electron microscopy (SEM) as previously described^26,27^. Bacterial cells in mid-log phase were diluted with PBS to obtain an OD_620_ of 0.05 and incubated at 37 °C for 0.25 h with peptide at a concentration of 0.5 × MIC. After incubation, the treated bacterial cells were centrifuged at 10,000 g for 10 min and washed 3 times with PBS, pH 7.2. Then, they were filtered through 0.22 µM mixed cellulose ester (MCE) membrane filters to retain the treated bacterial cells. Samples were pre-fixed by immersion into 2.5% (vol/vol) glutaraldehyde–PBS, then post-fixed with 1% OsO_4_ in DW. After washing 3 times, they were dehydrated using a graded ethanol series of 20%, 40%, 60%, 80% and 100%, 15 min in each dilution. After that, the samples were transferred into absolute ethanol 2 times, 15 min each time. Specimens were then dried and coated by platinum particle using Sputter Coater (Quorum Q150R ES; Quorum). Processed bacterial cells were observed using a scanning electron microscope (Hitachi SU8020; Hitachi, Japan).

### Transmission electron microscopy

The structural changes and integrity of bacterial membranes were investigated by transmission electron microscopy (TEM) as previously described^28^. Treatment of the bacterial samples was conducted in the same manner as described for the SEM treatment. After washing 3 times with PBS, overnight fixation with 2.5% glutaraldehyde was performed, and secondary fixation with 1% osmium tetroxide for 2 h. The fixed bacterial cells were washed thrice, followed by dehydration using a graded acetone series of 10%, 30%, 50%, 70%, 90% and 100% for 10 min each. After being placed in absolute acetone 2 times for 10 min each, infiltration of samples by 1:1, 1:2 and 2:1 mixture of acetone and epoxy resin for 3 h each was performed. Thereafter, the samples were transferred to pure epoxy resin 3 times for 3 h each time. Samples were embedded using a flat embedding mold. The castings were polymerized at 70 °C for 8 h. Then, ultrathin sections at a thickness of 70 nm were obtained using an ultramicrotome (Leica EM UC7; Leica). The samples were poststained with 5% uranyl acetate and lead citrate. Specimens were examined with a transmission electron microscope (Hitachi HT7700).

### Induction of resistance by serial passages at MIC

Serial 24-h passages of MIC were performed in 96-well plates in order to evaluate the development of resistance after long exposure to D-AP19 or meropenem, as previously described with some modification^29^. *A. baumannii* ATCC 19,606 was adjusted to approximately 10^6^ CFU/ml in MHB. The bacteria were treated with D-AP19 or meropenem at concentrations of 0.98 to 250 µg/ml, and 0.23 to 125 µg/ml, respectively. After 24 h incubation, bacterial suspensions of the wells at half-MIC concentration of tested agent were taken and washed 2 times with MHB to prepare the next initial-passage of bacteria. Twenty repeat passages were performed for each tested compound. Along with the test, a negative control was included: the MIC value of bacteria cultured in MHB-deionized water after 20 passages.

### Statistical analysis

The data from three independent experiments were exhibited as mean ± standard deviation (SD); differences with 95% confidence levels (p < 0.05) were considered statistically significant. One-way ANOVA with Tukey's Post Hoc Test analyzed via GraphPad PRISM software (version 7.0, GraphPad Software, California, USA) were applied to evaluate differences between control and tested groups.

## Results

### Peptide design and characteristics

**Figure 1:**
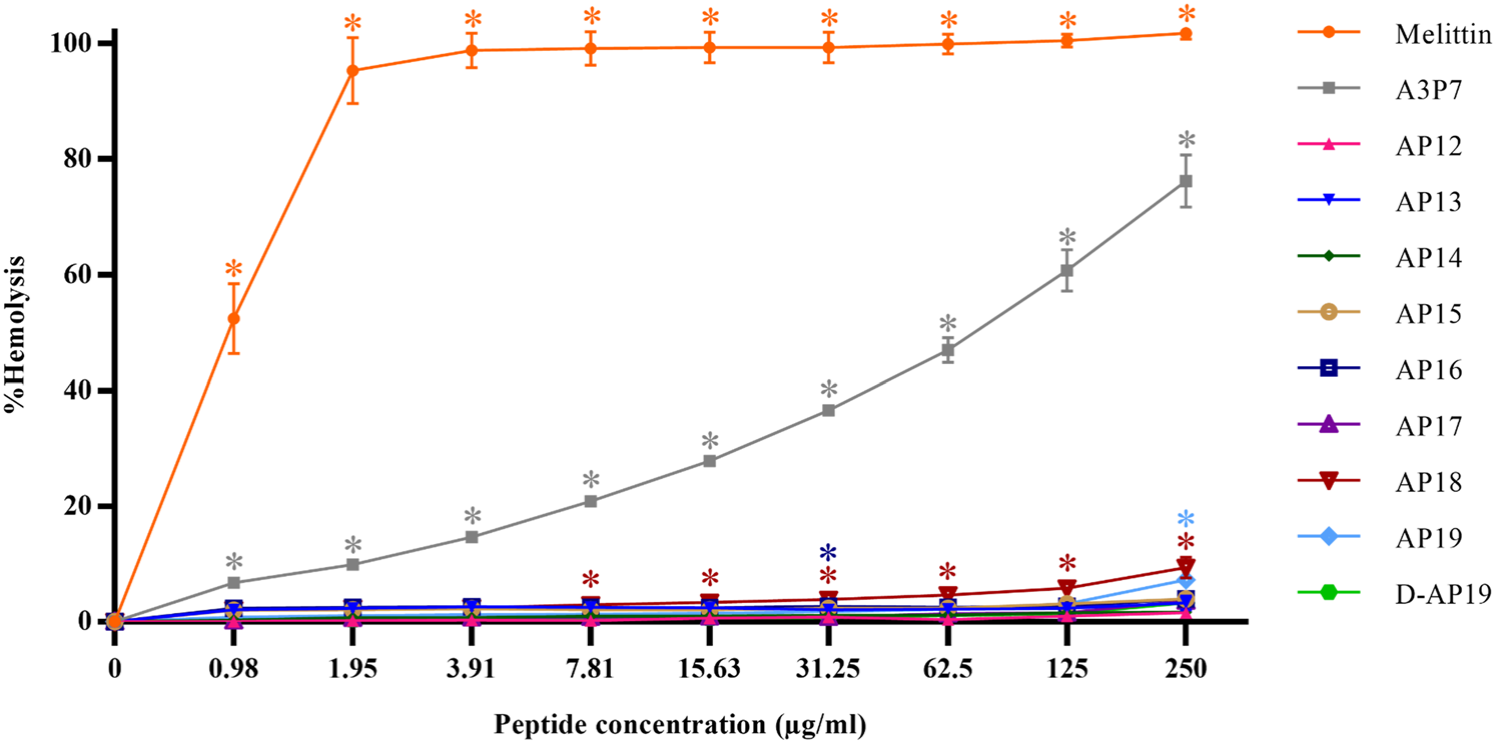
The hemolytic activity of parent peptide, A3P7, and its derivatives compared with that of melittin (a positive control). Three independent experiments were performed and the data are presented as mean ± SD. The statistical analyses utilized one-way ANOVA and Tukey’s test (GraphPad Prism7). Star indicates a significant difference from negative control (*p-value < 0.05).

The parent peptide, A3P7, was designed by flipping of the hybrid analogue peptide, P7A3, which was successfully modified from the sequence of cathelicidin and aurein^18^. A3P7 showed very high toxicity toward human red blood cells (Fig. 1) and therefore was serially truncated (from the C-terminal end) to obtain a peptide retaining potent antimicrobial potency but with reduced toxicity. As shown in Table 1, eleven derivatives were obtained and their physicochemical characteristics were in silico characterized. All truncated peptides were chemically synthesized for in vitro antimicrobial testing. Among the truncated derivatives, the 19-amino acid peptide, AP19, showed equal hydrophobicity to that of the parent peptide, A3P7, with a high positive net charge (+ 9). ESI–MS was used to confirm the molecular weights of peptides (Table 1). The theoretical molecular weight (MW) of each peptide was consistent with its measured MW. This suggested that peptide synthesis was successful.

**Table 1:** Amino acid sequence and physicochemical characteristics of parent peptide and truncated derivatives.

Peptide	Sequence	aa^a^	Theoretical MW^b^	Measured MW^c^	Net charge	Pho%^d^
A3P7	RLFRRVKKVAGKIAKRIWKILRR-NH_2_	23	2891.71	2891.71	+ 11	47%
AP19	RLFRRVKKVAGKIAKRIWK-NH_2_	19	2352.96	2353.00	+ 9	47%
AP18	RLFRRVKKVAGKIAKRIW-NH_2_	18	2224.79	2224.83	+ 8	50%
AP17	RLFRRVKKVAGKIAKRI-NH_2_	17	2038.58	2038.62	+ 8	47%
AP16	RLFRRVKKVAGKIAKR-NH_2_	16	1925.42	1925.46	+ 8	43%
AP15	RLFRRVKKVAGKIAK-NH_2_	15	1769.24	1769.27	+ 7	46%
AP14	RLFRRVKKVAGKIA-NH_2_	14	1641.06	1641.10	+ 6	50%
AP13	RLFRRVKKVAGKI-NH_2_	13	1569.99	1570.02	+ 6	46%
AP12	RLFRRVKKVAGK-NH_2_	12	1456.83	1456.86	+ 6	41%

^a^aa, number of amino acids.

^b^MW, molecular weight (g/mol) calculated by the website: https://pepcalc.com/.

^c^MW, molecular weight (g/mol) measured by mass spectroscopy (MS).

^d^Pho%, the percentage of hydrophobic residues.

### Candidate peptide AP-19 exhibited lowest GM value and highest therapeutic index

As shown in Table 2, the parent peptide, A3P7, exhibited strong antibacterial activity with MIC and MBC values ranging from 3.91 to 15.63 µg/mL against all tested strains of bacteria. Antibacterial activity of truncated peptides was found to be associated with the length of their amino acid sequence. The shortest truncated derivatives (AP12, AP13, and AP14) showed very low antibacterial activity against both Gram-negative and Gram-positive bacteria at MICs and MBCs of 31.25 to more than 250 µg/mL. The intermediate length peptides (AP15, AP16, and AP17) showed moderate inhibitory activity against all tested bacterial strains at MIC and MBC concentrations of 7.81 to more than 250 µg/mL. The peptides AP18 and AP19 showed the most potent antibacterial activity as demonstrated by having the lowest MIC and MBC values. Based on comparing geometric mean (GM) values (Table 2), AP19 had the greatest antimicrobial activity against both Gram-negative and -positive bacteria (had the lowest GM values).

**Table 2:** Antibacterial activity, minimum hemolytic concentration (MHC), geometric mean of MIC value (GM) and therapeutic index (TI) of hybrid peptide derivatives.

Bacterial strains	MIC (µg/ml)									
	A3P7	AP19	AP18	AP17	AP16	AP15	AP14	AP13	AP12	Melittin
**Gram-negative bacteria**										
*Acinetobacter baumanii* ATCC 19,606	7.81	7.81	7.81	31.25	62.5	62.5	> 250	> 250	> 250	3.91
*Escherichia coli* ATCC 25,922	7.81	3.91	7.81	15.63	31.25	31.25	125	125	125	3.91
*Escherichia coli* O157:H7 MT strain	15.63	15.63	15.63	125	125	62.5	250	250	250	7.81
*Pseudomonas aeruginosa* ATCC 27,853	7.81	3.91	3.91	15.63	62.5	125	250	> 250	> 250	7.81
*Salmonella Typhimurium* ATCC 13,311	7.81	1.95	3.91	7.81	15.63	31.25	62.5	62.5	125	3.91
*Shigella sonnei* ATCC 11,060	7.81	3.91	3.91	7.81	15.63	62.5	125	125	250	3.91
**Gram-positive bacteria**										
*Bacillus cereus* ATCC 11,778	7.81	7.81	7.81	62.5	250	> 250	> 250	> 250	> 250	7.81
*Listeria monocytogenes* 10403S	3.91	3.91	3.91	7.81	15.63	31.25	62.5	62.5	125	3.91
*Staphylococcus aureus* ATCC 25,923	7.81	7.81	7.81	62.5	125	250	> 250	> 250	> 250	15.63
*Staphylococcus epidermidis* ATCC 12,228	7.81	1.95	3.91	7.81	7.81	15.63	31.25	62.5	250	3.91
MHC^a^ (µg/ml)	1.95	> 250	> 250	> 250	> 250	> 250	> 250	> 250	> 250	< 0.98
GM^b^ (Gr.˗ strains)	9.11	7.16	7.16	23.44	52.09	62.5	218.75	250	260.42	5.21
Therapeutic index (TI)^c^ (Gr.˗ strains)	0.21	69.8	69.8	21.33	9.60	8.00	2.29	2.00	1.92	0.09
GM^d^ (Gr. + strains)	6.84	5.86	7.82	35.16	99.61	199.22	273.44	281.25	343.75	7.82
Therapeutic index (TI)^e^ (Gr. + strains)	0.29	85.32	63.98	14.22	5.02	2.51	1.83	1.78	1.45	0.06

^a^MHC, minimum hemolytic concentration, indicated the peptide concentration that caused 10% hemolysis of human red blood cells (hRBC). When there was less than 10% hemolysis detected at concentration of 250 µg/ml, a value of 500 µg/ml was applied to calculate the therapeutic index. When there was less than 10% hemolysis detected at concentration less than 0.98 µg/ml, a value of 0.49 µg/ml was applied to calculate the therapeutic index.

^b^GM (Gr.- strains) indicated the geometric mean of MIC value from all Gram-negative bacterial strains. When MIC was not detected at 250 µg/ml, a value of 500 µg/ml was applied to calculate the therapeutic index.

^c^Therapeutic index (Gr.- strains) is the ratio of the MHC to the geometric mean of MICs from Gram-negative bacterial strains.

^d^GM (Gr. + strains) indicates the geometric mean of MIC values from all Gram-positive bacterial strains. When MIC was not detected at 250 µg/ml, a value of 500 µg/ml was applied to calculate the therapeutic index.

^e^Therapeutic index (Gr. + strains) is the ratio of the MHC to the geometric mean of MICs from all Gram-negative bacterial strains.

The hemolytic activity of peptides against fresh hRBCs was determined at various concentrations (0.98 to 250 µg/ml) to indicate their toxicity on mammalian cells (Fig. 1). Melittin, the positive control, completely lysed hRBCs at a concentration of 3.91 µg/ml. The parent peptide (A3P7) had obvious hemolytic effects displayed in a concentration-dependent manner. Of truncated peptides, AP12 to AP19 showed a marked decrease in hemolytic effects on hRBCs at all concentrations, with MHC (minimum concentration that causes 10% hemolysis) greater than 250 µg/mL. These results highlighted an advantage of truncation, a significantly reduced toxicity of the peptides. The therapeutic index (TI) is an important parameter to identify a candidate peptide, reflecting a balance between efficacy and safety^30^. It was calculated as the MHC value divided by GM value (Table 2)^23^. For Gram-negative bacteria, AP18 and AP19 exhibited the highest TI values of the designed peptides. For Gram-positive bacteria, AP19 showed the highest TI value. The TI value of AP19 against Gram-negative and -positive bacteria was 332 and 294 times higher, respectively, than that of A3P7 parent peptide.

### D-AP19 displayed potent antibacterial activity toward MDR and XDR *A. baumannii* along with improved stability

AMPs have failed in clinical trials due to their low stability when exposed to proteolytic enzymes and human plasma. Due to chirality mismatch with active sites of enzymes, D-enantiomers are usually resistant to proteolysis by endogenous enzymes, especially proteases^31^. Thus, to increase its stability toward proteolytic enzymes peptide AP19 was further modified by D-amino acid substitution and designated as D-AP19. It retained the same MIC and MBC values against *A. baumannii* (a first priority pathogen on the WHO list) in contrast to AP19 (Table 3). The MIC and MBC values of AP19 against *A. baumannii* ATCC 19,606 increased more than 32-fold (from 7.81 to more than 250 µg/mL) in the presence of trypsin and proteinase K, indicating a complete loss of AP19 anti-microbial activity (Table 4). D-AP19 displayed tolerance to trypsin and proteinase K as its MIC was not changed (7.81 µg/mL) in the presence of these two proteases when compared to untreated peptide. With pepsin treatment, the MICs of AP19 and D-AP19 were fourfold (from 7.81 to 31.25 µg/mL) and twofold (from 7.81 to 15.63 µg/mL) increased, respectively. These finding suggested that D-AP19 exhibited more stability to proteolytic enzymes than did AP19.

**Table 3:** Minimum inhibitory concentration (MIC) and minimum bactericidal concentration (MBC) of AP19 and D-AP19 compared to commonly used antibiotics against eight strains of MDR and XDR *A. baumannii* clinical isolates.

Bacterial strains	Source	MIC (MBC) (µg/ml)					
		AP19	D-AP19	Meropenem	Levofloxacin	Minocycline	Colistin
**Standard strain**							
ATCC 19,606	ATCC	7.81 (7.81)	7.81 (7.81)	0.98(0.98)	≤ 0.49(≤ 0.49)	≤ 0.49(1.95)	0.98(0.98)
**Clinically isolated drug-resistant *A. baumannii***							
A-8.2–1 (XDR)	Blood	3.91(3.91)	7.81(7.81)	62.5(62.5)	3.91(3.91)	0.98(31.25)	1.95(1.95)
B-8.1–1 (MDR)	Respiratory tract	7.81(7.81)	7.81(7.81)	0.98(15.63)	≤ 0.49(1.95)	≤ 0.49(7.81)	1.95(1.95)
B-8.1–2 (MDR)	Respiratory tract	7.81(7.81)	15.63(15.63)	125(125)	125(250)	0.98(15.63)	0.98(0.98)
B-8.2–2 (XDR)	Respiratory tract	7.81(7.81)	7.81(7.81)	125(125)	7.81(7.81)	3.91(31.25)	1.95(1.95)
C-8.1–2 (MDR)	Sterile site	3.91(3.91)	3.91(3.91)	125(125)	31.25(31.25)	3.91(31.25)	1.95(1.95)
C-8.2–1 (XDR)	Sterile site	7.81(7.81)	7.81(7.81)	125(250)	3.91(7.81)	1.95(31.25)	0.98(0.98)
D-10.1–1 (MDR)	Urine	7.81(7.81)	7.81(7.81)	62.5(62.5)	3.91(7.81)	0.98(31.25)	0.98(0.98)
D-10.1–2 (MDR)	Urine	3.91(3.91)	7.81(7.81)	62.5(62.5)	31.25(31.25)	1.95(3.91)	1.95(1.95)

**Table 4:** The antimicrobial activity (MIC and MBC) (µg/ml) of AP19 and D-AP19 against *A. baumannii* ATCC 19,606 in the presence of human plasma of *A. baumannii* and proteolytic enzymes.

Peptides	Control^a^	Human plasma	Proteolytic enzymes^b^		
			Pepsin	Trypsin	Proteinase K
AP19	7.81(7.81)	250(250)	31.25(31.25)	> 250(> 250)	> 250(> 250)
D-AP19	7.81(7.81)	31.25(31.25)	15.63(15.63)	7.81(7.81)	7.81(7.81)

^a^Control MIC was evaluated in the absence of human plasma and proteolytic enzymes.

^c^The final concentration of all proteolytic enzymes (pepsin, trypsin and proteinase K) was 1 mg/ml.

The nosocomial pathogen *A. baumannii* is responsible for various types of infections in the healthcare setting, including pneumonia, bacteremia and meningitis^32^. Its threat is growing as indicated by steady increase in prevalence of MDR or XDR *A. baumannii* over the past decade^32^. In this study, D-AP19 showed high antibacterial activity against all MDR and XDR clinical isolates of *A. baumannii* with MICs and MBCs ranging from 3.91 to 15.63 µg/mL (Table 3)*.* For comparison, the antibacterial activities of meropenem, levofloxacin, minocycline and colistin, the commonly used antibiotics for in-hospital *A. baumannii* infections, were also examined against these drug-resistant bacteria. The antibacterial activity of AP19 and D-AP19 against meropenem-resistant *A. baumannii* (MIC value of 3.91 to 15.63 µg/mL), was 16 to 32 times higher than that of meropenem (MIC value of 62.5 to 125 µg/mL).

Some components of human plasma, such as binding or blocking proteins and peptidases, can inactivate peptides or degrade their amino acid sequences^33^. After incubation with human plasma, the MIC and MBC values of AP19 were 32-fold increased from 7.81 to 250 µg/mL, whereas the MICs of D-AP19 were only twofold increased (from 7.81 to 15.63 µg/mL) (Table 4). These results indicated that D-AP19 was more stable in human plasma than was AP19.

### D-AP19 displayed low toxicity to mouse fibroblast cells and low hemolytic activity to human RBCs

**Figure 2:**
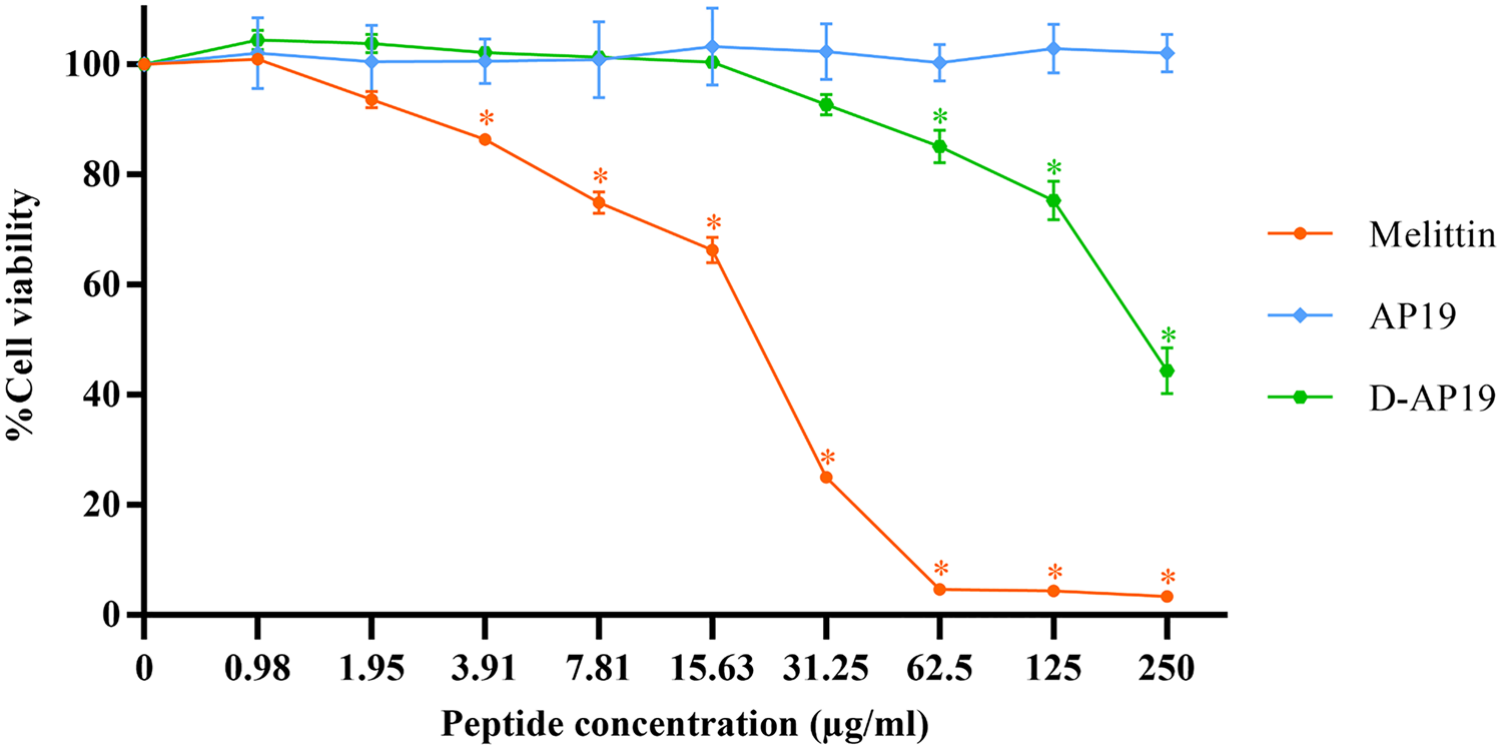
The cytotoxicity of AP19 and D-AP19 against L929 mouse fibroblast cells compared with that of melittin (a positive control). Three independent experiments were performed and the data are presented as mean ± SD. The statistical analyses utilized one-way ANOVA and Tukey’s test at *p*-value < 0.05 (GraphPad Prism7). Star indicates a significant difference from negative control (*p*-value < 0.05).

The cytotoxic activity of AP19 and D-AP19 was investigated by measuring viability of L929 mouse fibroblast cells after exposure to peptide. The membranolytic melittin was included in this test as a positive control. Melittin had robust cytotoxicity in a concentration-dependent manner, indicated by the decrease of cell viability to less than 5% at a peptide concentration of 62.5 µg/mL (Fig. 2). In contrast, AP19 showed no toxicity as indicated by 100% cell viability for all tested peptide concentrations. At 1 × and 2 × MIC of D-AP19, 100% cell viability was observed. In contrast, the cell viability of D-AP19 at peptide concentrations of 62.5, 125 and 250 µg/mL was significantly greater than that of the negative control (*p* > 0.05). But based on the ISO standards^34^, D-AP19 (at concentrations of 62.5 and 125 µg/mL) was not considered a cytotoxic agent since cell viability was more than 70%. The hemolytic activity of D-AP19 was decreased compared with AP19. Hemolysis was 7.25% and 3.28% at a concentration of 250 µg/mL for AP19 and D-AP19, respectively. At the MIC of D-AP19 against *A. baumannii* ATCC 19,606, hemolysis was only 0.58%.

### D-AP19 retained its activity in the presence of physiological salts

The MICs of AP-19 and its D-enantiomers for *A. baumannii* ATCC 19,606 were examined in the presence of 150 mM NaCl, 4.5 mM KCl, 1 mM MgCl_2_, 6 µM NH_4_Cl, 8 µM ZnCl_2_, 4 µM FeCl_3_ and 2.5 µM CaCl_2_ (Table 5). AP19 still possessed effective antibacterial activity against *A. baumannii* in MHB with KCl, MgCl_2_, NH_4_Cl, ZnCl_2_, FeCl_3_, and CaCl_2_ (MIC of 7.81 µg/mL), and its MIC value was fourfold increased (to 31.25 µg/mL) in the presence of Na^+^. The MIC of D-AP19 was increased further (to about eightfold) in the presence of Na^+^. Peptide D-AP19 appeared to be moderately resistant to most of physiologic serum cations [monovalent (K^+^, NH_4_^+^), divalent (Mg^2^^+^, Ca^2^^+^, Zn^2^^+^) and trivalent (Fe^3^^+^)] with MIC values of 7.81 to 15.63 µg/mL. These results suggested that both L- and D-form of AP19 peptide exhibited antibacterial activity despite the presence of most physiologic salts. But of note, NaCl reduced the antibacterial activity of both peptides.

**Table 5:** The antimicrobial activity (MIC and MBC) (µg/ml) of AP19 and D-AP19 against *A. baumannii* ATCC 19,606 in the presence of serum salts at physiological concentrations.

Peptides	Control^a^	Physiological salts^b^						
		NaCl	KCl	MgCl_2_	NH_4_Cl	ZnCl_2_	FeCl_3_	CaCl_2_
AP19	7.81(7.81)	31.25(31.25)	7.81(7.81)	7.81(7.81)	7.81(7.81)	7.81(7.81)	7.81(7.81)	7.81(7.81)
D-AP19	7.81(7.81)	62.5(62.5)	15.63(15.63)	7.81(7.81)	7.81(7.81)	15.63(15.63)	15.63(15.63)	15.63(15.63)

^a^Control MIC was evaluated in the absence of serum salts.

^b^The final concentrations of NaCl, KCl, MgCl_2_, NH_4_Cl, ZnCl_2_, FeCl_3_, and CaCl_2_ were 150 mM, 4.5 mM, 1 mM, 6 µM, 8 µM, 4 µM and 2.5 µM, respectively.

### D-AP19 and its L-enantiomers formed an α-helical amphipathic conformation in membrane mimetic environments

**Figure 3:**
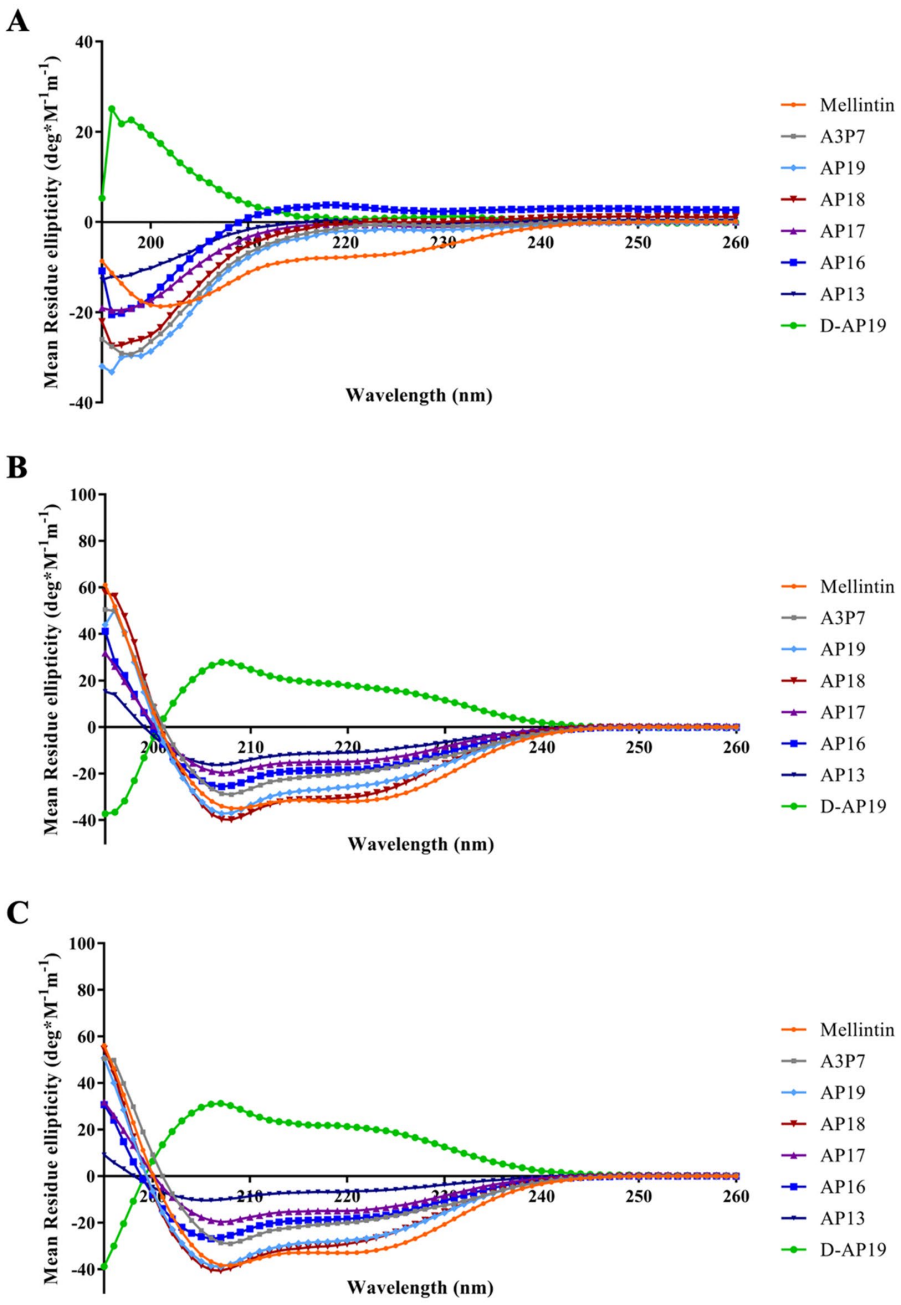
The circular dichroism spectra of melittin, A3P7, AP19, AP18, AP17, AP16, AP13 and D-AP19 in PBS (**A**), 30 mM SDS micelles in PBS (**B**), and 50% (v/v) TFE in PBS (**C**). Peptides were dissolved in each solution, filled in a 0.1 cm path length rectangular quartz cell and measured at 25 °C by Jasco-815 spectropolarimeter. Three scans of CD spectra were analyzed in the wavelength range of 190 to 260 nm at scanning speed of 10 nm/min.

The secondary structure of peptides in PBS as well as in the membrane mimetic environments (30 mM SDS micelles in PBS and 50% TFE in PBS) was determined by CD spectroscopy. The CD spectra analyses were interpreted in accordance with published data^35,36^. As shown in Fig. 3, the spectra of all peptide derivatives (A3P7, AP13, AP16, AP17, AP18, AP19 and D-AP19) in PBS showed obvious negative signals at around 200 nm [characteristic of a random coil (or unordered) structure], while melittin showed the weak signal of an α-helical structure. In 30 mM SDS and 50% TFE, melittin and A3P7 formed α-helical conformations with maximum signals at 192 nm and two minimum signals around 208 and 222 nm. The truncated peptides (AP13, AP16, AP17, AP18 and AP19) formed α-helical conformations in 30 mM SDS micelles. In 50% TFE, peptides AP16, AP17, AP18 and AP19 adopted α-helical configurations, while AP13 formed a weak α-helical structure (Fig. 3). D-AP19 displayed characteristic signals for a random coil structure in PBS, and strong α-helical configurations in 30 mM SDS micelles and 50% TFE. The CD spectra of D-enantiomers of AP19 were mirror images of those of its L-enantiomer. These results indicated that A3P7, AP19, AP18 and AP17, as well as D-AP19, transformed from an unordered random coil structure in aqueous environment to an α-helical amphipathic conformation in membrane mimetic environments.

### D-AP19 exhibited rapid killing of *A. baumannii*

**Figure 4:**
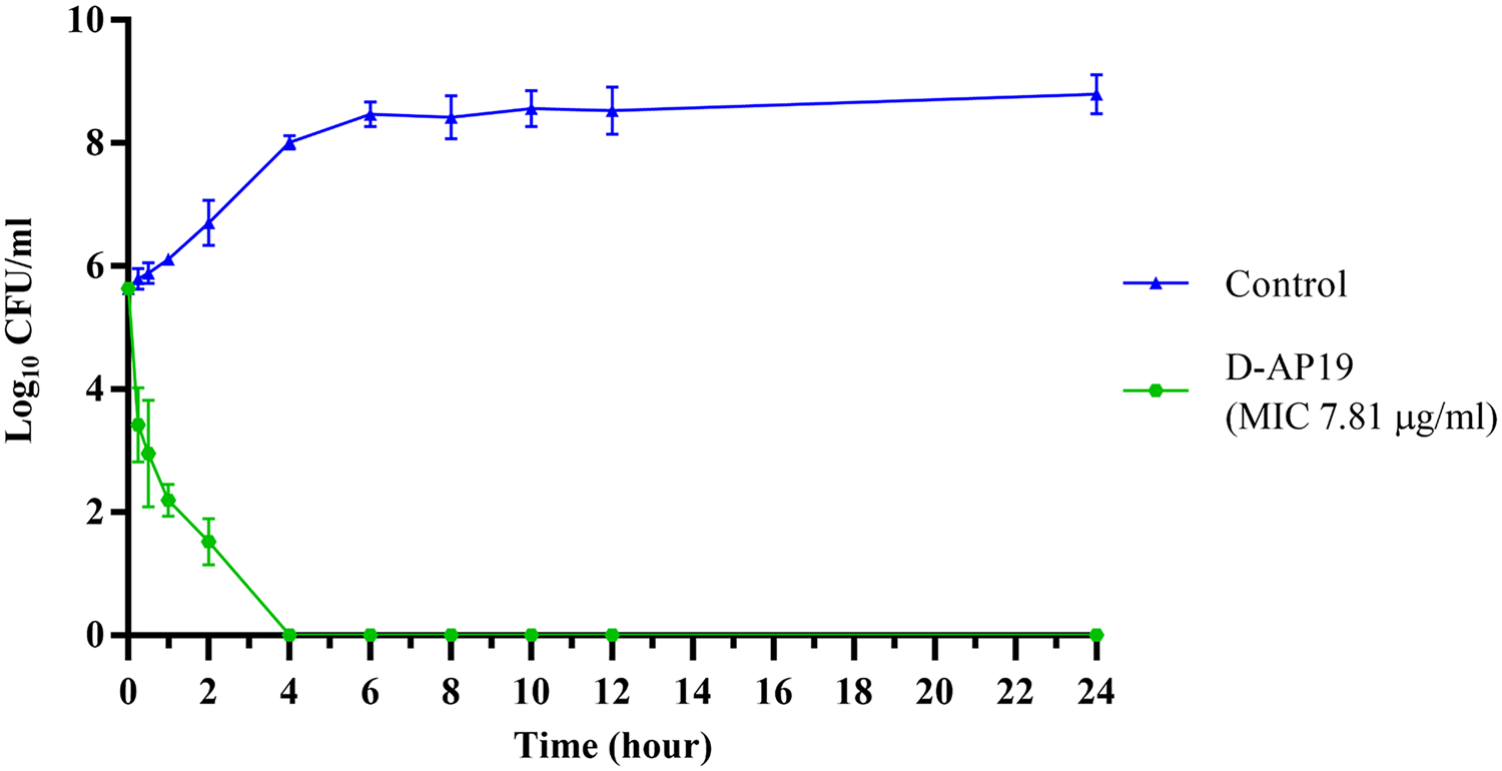
Time-kill kinetics of D-AP19 at 1 × MIC against *A. baumannii* ATCC 19,606 for 24 h. Control (blue line) indicates untreated bacteria grown in MHB-DI solution. Green line indicates *A. baumannii* ATCC 19,606 treated with D-AP19 at 1 × MIC.

The time course for bactericidal activity of D-AP19 against *A. baumannii* ATCC 19,606 was determined (shown in Fig. 4). Compared to growth of the bacterial negative control, D-AP19 decreased cell viability of *A. baumannii* more than 10^2^, 10^3^ and 10^4^ CFU/mL within 0.25, 0.5 and 1 h, respectively. Within 4 h, D-AP19 completely killed all *A. baumannii* and no regrowth was observed after 24 h of observation. As seen in Table 3, the MIC of D-AP19 against each of the standard and drug-resistant strains of *A. baumannii* was equal to its MBC. Therefore, complete killing of the bacterial inoculum after 4 h was observed at the MIC concentrations. Therefore, D-AP19 demonstrated rapid killing and time-dependent bactericidal activity.

### D-AP19 induced bacterial membrane depolarization and permeabilization leading to cell death

**Figure 5:**
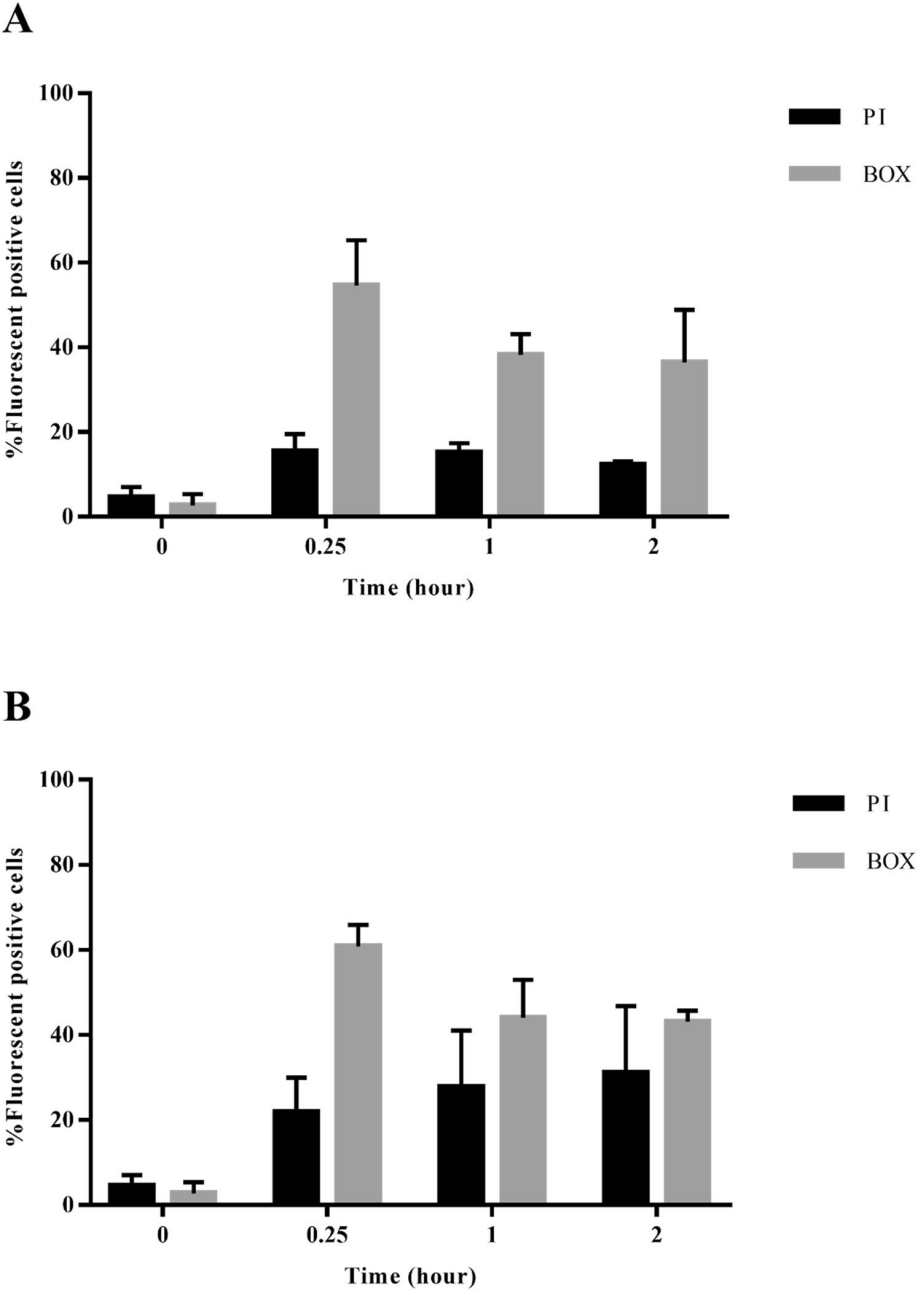
Percentage of fluorescent positive cells stained with PI or BOX after treatment with D-AP19 for 2 h. *A. baumannii* ATCC 19,606 was treated with half MIC (**A**) or 1 × MIC (**B**), and stained with a fluorescent dye.

The induction of cytoplasmic membrane depolarization and permeabilization of *A. baumannii* ATCC 19,606 was evaluated after treatment with D-AP19 at 1 × MIC) using two fluorescent dyes (PI and BOX). PI intercalates in the nucleic acids of bacterial cells with permeabilized membranes, and becomes fluorescent. BOX, a plasma membrane potential-sensitive dye, binds to lipid-rich components of depolarized cells and displays enhanced fluorescence. As shown in Fig. S1, unstained cells were utilized to set gates for the negative population. Without D-AP19 treatment (or 0 h treatment), 95 ± 2% and 97 ± 3% of *A. baumannii* cells were stained by neither PI nor BOX, respectively, implying intact cell membranes (Fig. 5). For positive control, a high degree of membrane permeabilization and depolarization was induced by heating cells at 70 °C (a membrane damage temperature) for 30 min; this resulted in 82 ± 3% and 68 ± 17% cell positivity by PI and BOX, respectively (Fig. S2). D-AP19 at 0.5 × MIC induced membrane permeabilization (15 ± 4%, 15 ± 2% and 12 ± 1%) and depolarization (55 ± 10%, 38 ± 4% and 37 ± 12%) after 0.25, 1 and 2 h treatments, respectively (Fig. 5). At 1 × MIC, D-AP19 rapidly induced membrane permeabilization (22 ± 8, 28 ± 13 and 31 ± 15% in 0.25, 1 and 2 h, respectively) and depolarization (61 ± 5%, 44 ± 9 and 43 ± 2% in 0.25, 1 and 2 h, respectively). These results suggested that D-AP19 can damage the membrane of *A. baumannii* by both permeabilization and depolarization.

### Ultrastructural evidence of D-AP19’s disruption and distortion of bacterial cell membranes

**Figure 6:**
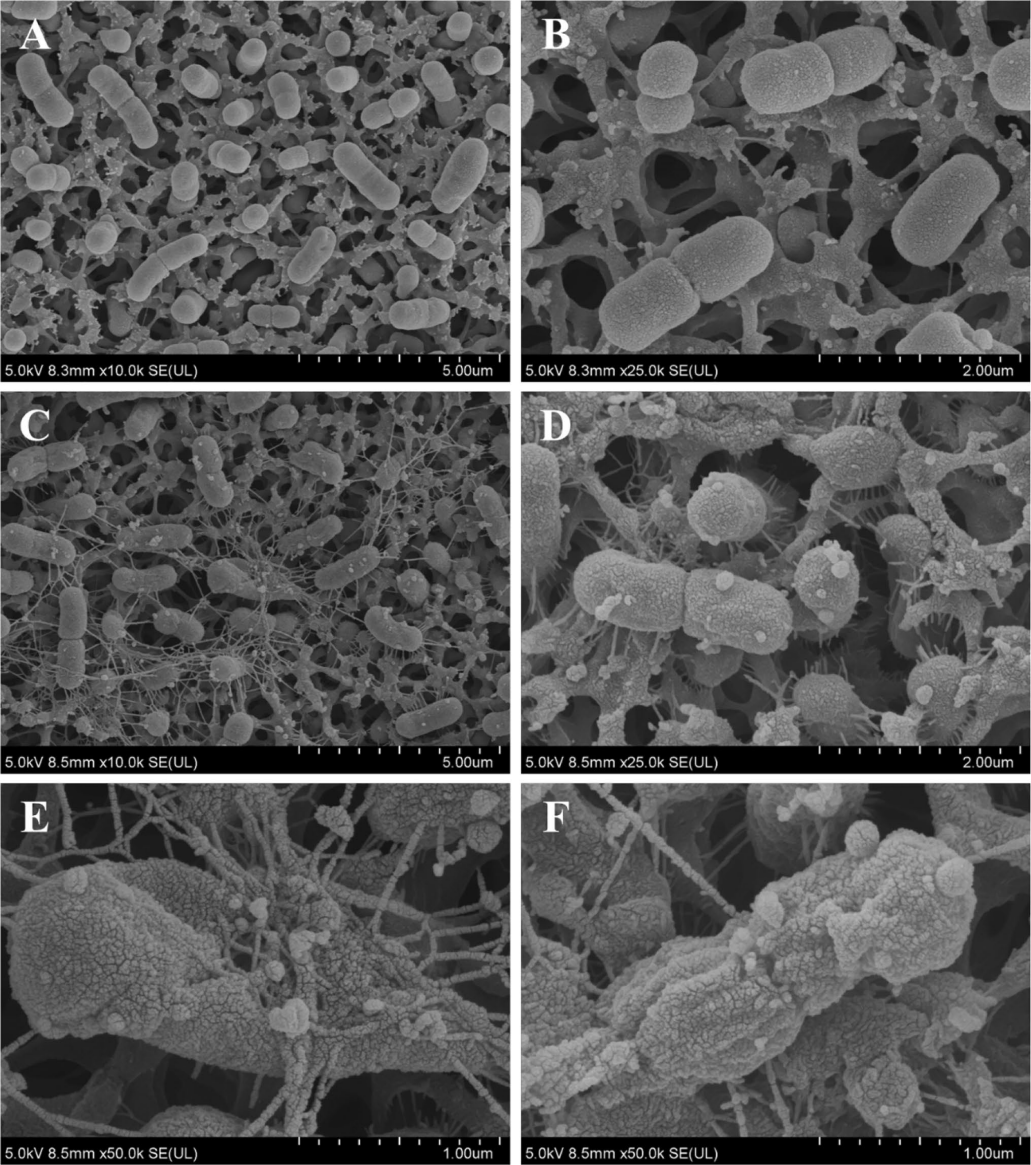
Scanning electron microscopic micrographs of *A. baumannii* ATCC 19,606 treated with D-AP19. Untreated bacterial cells (**A**), (**B**) and treated with half MIC of D-AP19 for 15 min (**C**)–(**F**).

Morphologic changes of *A. baumannii* ATCC 19,606 was directly observed using SEM. On untreated bacterial cells the membrane surfaces were smooth and intact (Fig. 6A and B). On the contrary, membranes of bacterial cells treated with 0.5 × MIC of D-AP19 for 15 min were significantly damaged. Rough surfaces and shrinkage of bacterial cells were observed (Fig. 6E,F). Multi-blebbing of many cells with pili-like structures were obvious (Fig. 6C,D), as well as distortion and disruption of bacterial cell membranes (Fig. 6E,F).

**Figure 7:**
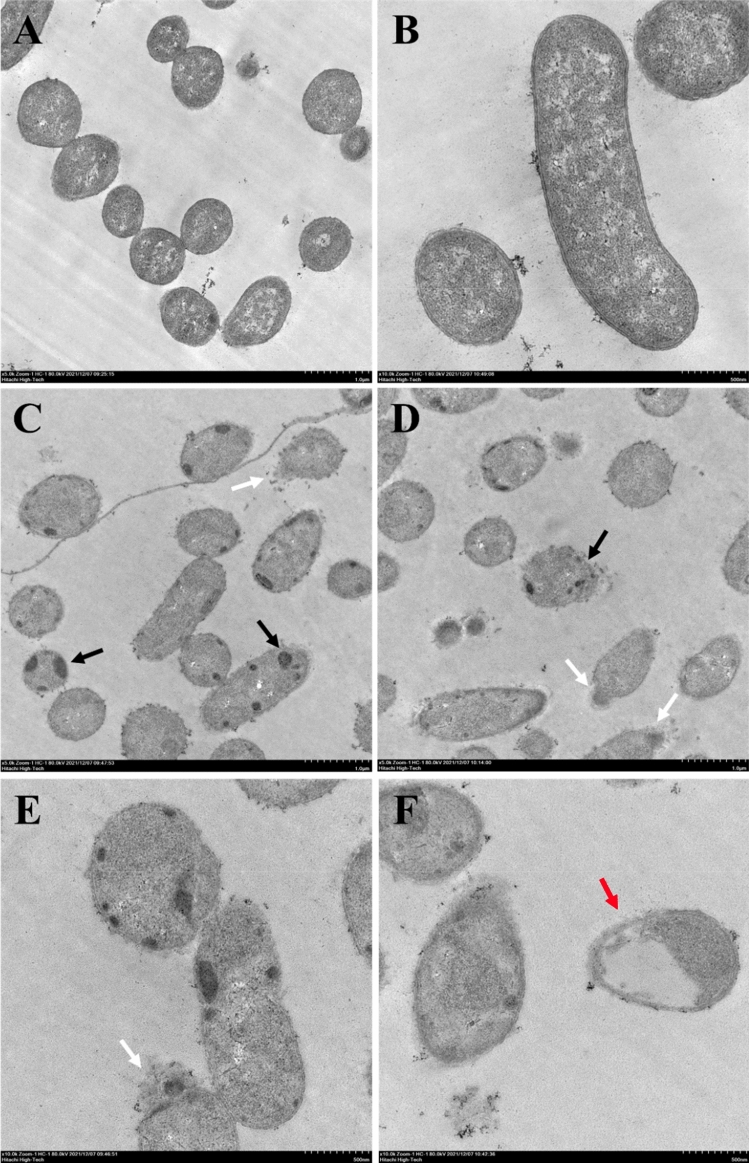
Transmission electron microscopic micrographs of *A. baumannii* ATCC 19,606 treated with D-AP19. Untreated bacterial cells (**A**), (**B**) and cells treated with half MIC of D-AP19 for 15 min (**C**)–(**F**). Black, white and red arrows indicate inclusion bodies, intracellular leakage and ghost cells in bacterial cells, respectively.

The effects of D-AP19 on the morphology and intracellular contents of *A. baumannii* ATCC 19,606 were also assessed by using TEM. *A. baumannii* grown in PBS without D-AP19 (negative control) displayed intact cell membranes and clearly visible cell walls (Fig. 7A and B). After 15 m incubation with D-AP19 (0.5 × MIC), ultrastructural alterations were observed, including obvious clumped material attached to inner membranes, apparent cytoplasmic clear zones, blurred cell membranes and intracellular leakage (Fig. 7C–F).

### D-AP19 did not induce resistance in *A. baumannii * in vitro

**Figure 8:**
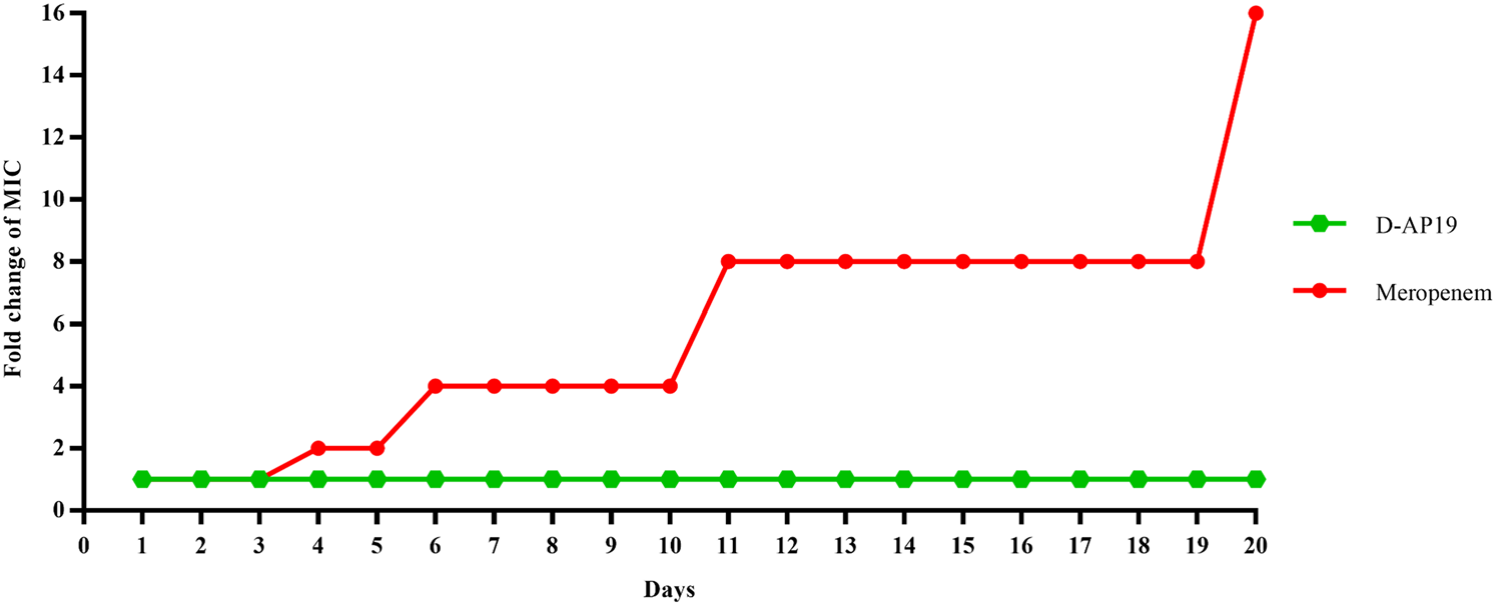
Fold changes of MICs of D-AP19 and meropenem against *A. baumannii* ATCC 19,606. The serial passages of bacteria were grown for 20 days in the presence of test compounds at different concentrations. The MIC and MBC values were determined after each passage.

The high propensity of antibiotic therapy to induce bacterial resistance is a major obstacle to continued efficacy of antibiotics, harming healthcare D-AP19 (0.5 × MIC) or meropenem was evaluated through 20 serial passages by broth microdilution assay (Fig. 8). Bacteria serially grown in drug-free medium for 20 passages were used as negative controls. The MICs of D-AP19 and meropenem against these negative control bacteria were equal to the MICs at the first passage, suggesting that *A. baumannii* ATCC 19,606 did not develop resistance in these non-drug conditions. At day 4, 6 and 11, MICs of meropenem were 2-, 4- and eightfold increased, respectively. After passage 20, meropenem exhibited a 16-fold increase in MIC compared to the first passage, resulting in a final MIC of 15.63 µg/mL (a 15-fold increase). Remarkably, D-AP19 antimicrobial activity did not decrease over the 20 passages, retaining an MIC value of 7.81 µg/mL.

## Discussion

Nosocomial bacterial infection is an important cause of morbidity and mortality among patients with serious health problems^2,37^. According to a WHO announcement, carbapenem-resistant *Acinetobacter baumannii* is classified as a priority pathogen which urgently needs new and effective therapeutic agents^3^. Antimicrobial peptides, cationic peptides with innate immune properties, possess multi-action mechanisms against pathogenic bacteria, including membrane attack, intracellular alteration and/or immunomodulation^9^. They have a low propensity to induce bacterial resistance and can kill drug-resistant bacteria^8^. Although these advantages are well-known, AMPs also have some weaknesses. For instance, they may be toxic to human cells and lose activity in the presence of proteolytic enzymes, salts, serum and/or plasma^9^. But of note, amino acid sequence modifications can overcome these shortcomings and generate potentially successful peptide-based drugs. Thus, the design and modification of membrane-penetrating AMPs is a prominent strategy to obtain novel antimicrobial agents with satisfactory efficacy and toxicity features.

We previously reported a hybrid peptide, a modified α-helix cathelicidin (P7) and aurein (A3) designated as P7A3, with promising antimicrobial activity and potential applications^18^. In the present study, a novel hybrid peptide (A3P7) was obtained using a flipping technique or reverse conjugation, in the hybridizing of A3 with P7 at the C-terminus. This peptide had imperfect amphipathicity, high positively charge and hydrophobicity, and exhibited broad-spectrum antibacterial activity against both Gram-negative and -positive bacteria comparable to that of P7A3, along with moderate hemolytic activity (compared to that of melittin). The active site of α-helical AMPs is mostly located at the N-terminus which normally interacts with and penetrates through the bacterial membrane^38^. To reduce sequence length and toxicity against human red blood cells, A3P7 was sequentially truncated at the C-terminus. Among the resulting truncated peptides, AP19 showed improved antimicrobial activity against some bacterial strains, with no increase in hemolytic and cytotoxic activity. These findings suggested that C-terminal truncation of amino acids at positions 20 to 23 could improve antibacterial activity and reduce the hemolytic activity, similar to the results of P5 and its truncated derivatives^25^. The longer peptides might have reduced antibacterial activity, because reaching the cytoplasmic membrane of bacteria is more difficult^39^. Moreover, they are usually more hemolytic than their shorter derivatives^9^. Multiple physiologic characteristics of AMPs, including number of amino acid residues (size), net charge, hydrophobicity, amphipathicity and helicity, are responsible for their antimicrobial and hemolytic activities^9,40^. But modification of AMPs has complicated effects, since changing one factor will affect others, and so multiple activities must be monitored as changes are made^41^.

The therapeutic index is a useful parameter in identifying candidate peptides, as it reflects a balance between the efficacy and safety of candidate agents^30^. Higher TI values indicate a peptide’s favorable safety and promising efficacy. Compared to A3P7, TI values of all truncated peptides were increased as a result of reduced hemolytic activity. The truncated peptide AP19 exhibited the highest TI value against both Gram-negative and Gram-positive bacteria with the values of 69.8 and 85.32, respectively. This resulted from strong antibacterial activity and weak hemolytic activity when compared to that of the parent peptide A3P7, reflecting greater therapeutic benefit without increased toxicity. These results suggested that C-terminal truncation improved the selectivity of peptides toward bacterial cells more than hRBCs. The successful development of AP19, as indicated by its high antibacterial activity and low hemolytic activity, opened interesting opportunities for AMP development and practical application.

An important obstacle responsible for past failures of AMPs in clinical trials is their loss of antibacterial activity in the presence of proteolytic enzymes and human plasma^42^. AP19 showed low stability in pepsin, trypsin, proteinase K and human plasma. Due to chirality mismatches with active sites of enzymes, D-enantiomers are usually resistant to proteolysis by endogenous enzymes, especially proteases^31^. To improve the stability of our peptide, AP19 was further modified by D-amino acid substitution (designated as D-AP19). The antimicrobial activity of D-AP19 against *A. baumannii* ATCC 19,606 was similar to that of AP19, suggesting D-amino acid incorporation did not affect this activity of the peptide, a finding similar to that of other studies^43,44^. D-AP19 retained potent antibacterial activity after exposure to all tested commercial proteases (pepsin, trypsin and proteinase K) and human plasma with MICs from 7.81 to 31.25 µg/mL. This was consistent with other research that D-amino acid substitution is an effective strategy to improve peptide stability against proteases and human plasma^43,44^.

WHO has announced an urgent need for development of new antimicrobial agents to fight against MDR bacteria, especially multidrug-resistant *A. baumannii*^3^. AP19 and D-AP19 exhibited the same level of antibacterial activity against both reference and resistant strains of MDR and XDR *A. baumannii*, whereas traditional antibiotics (meropenem and levofloxacin) required higher concentrations to kill these bacteria. Thus, our designed peptide was effective against resistant strains of clinically isolated *A. baumannii* and its antibacterial activity was not significantly affected by the bacterial resistance phenotype^43–45^.

Others have reported that physiologic concentrations of serum salts diminish the antimicrobial activity of several peptides, and that this is due to the interruption of the electrostatic interactions between positively charged AMPs and negatively charged microbial membranes in the presence of salt ions^46^. AP19 retained antibacterial activity in the presence of all tested serum ions, except Na^+^. Peptide D-AP19 retained its antibacterial activity in the presence of Mg^2^^+^ and NH_4_^+^, while its activity was slightly weakened by K^+^, Zn^+^, Ca^2^^+^ and Fe^3^^+^. In the presence of Na^+^, the antibacterial activity of AP19 and D-AP19 was greatly decreased. The results revealed that D-amino acids substitution of AP19 could not enhance the peptide stability in the presence of serum salts. RR4 and its D-enantiomer showed similar antimicrobial activities in the presence of Na^+^, Ca^2^^+^ and Mg^2^^+^ ions^43^.

The toxicity of D-AP19 against hRBCs and L929 mouse fibroblast cells was compared to that of AP19. D-AP19 showed slightly decreased hemolytic activity, thus a better safety profile than AP19. Similarly, the D-forms of Bac and GL13K peptides have less hemolytic activity than their L-forms^45,47^. On the contrary, D-AP19 at high concentrations showed greater toxicity on L929 cells than did its L-form. Similarly, the D-enantiomers (Pro9-3D and Pro10-1D) of peptide Pro9-3 and Pro10-1 showed greater toxicity against mouse macrophage RAW264.7 cells than did their L-enantiomers^48^. These results suggested that D-AP19 was not toxic against human red blood cells but was against mouse cells.

The helix forming ability of peptides in membrane mimetic environments was correlated with their antimicrobial activity^49,50^. Melittin, A3P7, AP19, AP18, AP17, AP16 and D-AP19 formed α-helical amphipathic structures in both 30 mM SDS and 50% TFE with high and moderate antimicrobial activity. AP13 failed to form a helical conformation in 50% TFE, but did so in 30 mM SDS; these had low antimicrobial activity. From previous studies, C-terminal truncated derivatives of P5 peptide (P5-CT2) lose the ability to form α-helices in 30 mM SDS, but retain this in 50% TFE; antimicrobial activity is lower than that of P5^25^. In parallel, the helical content of peptides when interacting with 50% TFE was correlated with toxicity against hRBCs^49^. A3P7 possessed 58.4% α-helical content and showed an MHC value of 1.95 µg/mL. Previous studies revealed that the hemolytic activity of peptides generally decreased with reduction of peptide helicity^22,49^.

AMPs have multiple mechanisms of action aimed at multiple targets in bacteria, including cytoplasmic membranes and intracellular targets^51^. Most AMPs interact with and disrupt bacterial membranes, causing membrane depolarization and permeabilization, and leading to cell dead^10,52^. However, the exact mechanism of these actions is unclear^52^. Membrane depolarization and permeabilization after treatment by D-AP19 were analyzed using BOX and PI fluorescent dyes, respectively. The results showed that these effects were not time dependent. Within 15 min, D-AP19 had induced bacterial membrane depolarization and permeability. This directly correlated with D-AP19’s fast killing at MIC, with approximately 30–40% of *A. baumannii* killed within 15 min. The SEM and TEM studies showed that D-AP19 damaged *A. baumannii* via membrane-based mechanisms and leakage of intracellular contents, with additional unknown mechanisms resulting in the formation of inclusion bodies which accumulated at the inner bacterial membranes. The membrane depolarization and permeabilization is one of killing mechanism of D-AP19. Many AMPs have been reported to depolarize and permeabilize the membranes of *A. baumannii*, resulting in intracellular leakage^44,53^. The limitation of this study was that the mechanism(s) of action of D-AP19 toward intracellular target in *A. baumannii* ATCC 19,606 was not clearly identified.

In the clinical use of antibiotics to treat nosocomial infection, development of bacterial resistance interrupts the efficiency of treatment. The serial incubation of bacteria in half-MIC of peptide is a widely used method of inducing resistance. Using the method, D-AP19 retained its antibacterial activity at 7.81 µg/mL without increase through 20 culture passages, while under the same conditions meropenem showed an increase of MIC. These results are similar to those with AA139 and ZY4 peptides^29,54^. Induction of resistance to antibiotics is often associated with a single mechanism of action^54^. D-AP19, with its multi-mechanisms of action, showed a very low propensity to induce resistance, and so might be an effective way to combat the development of resistant bacteria^54^.

## Conclusion

This study emphasized the potential to develop a protease tolerant, membrane-active peptide (D-AP19), as a therapeutic agent especially against multi- or extensively-drug resistant *A. baumannii* with no toxicity to human cells. In addition, the antibacterial activity of D-AP19 was not affected by the resistance phenotype of the target bacteria. Our candidate peptide (D-AP19) rapidly killed bacteria through interactions with membrane targets and forming α-helical structures. Subsequently, membranes became depolarized and permeable, leading to leakage of intracellular contents and membrane disruption, and ultimately cell death. Importantly, D-AP19 also displayed a low tendency to induce bacterial resistance. This study exploited the advantages of peptide modification for improvement of AMPs in terms of antibacterial activity, safety and stability*.* With further study of efficacy and safety carried out in vivo, peptide D-AP19 might be shown to have potential for human therapy, and join the challenging fight against MDR or XDR *A. baumannii*.

## Supplementary Information

Supplementary Information.

## Data Availability

All of the data produced throughout the research are contained in this article and the supplementary information file.

## References

[1] Molton JS, Tambyah PA, Ang BS, Ling ML, Fisher DA (2013). The global spread of healthcare-associated multidrug-resistant bacteria: a perspective from Asia. Clin. Infect. Dis..

[2] Khan HA, Baig FK, Mehboob R (2017). Nosocomial infections: epidemiology, prevention, control and surveillance. Asian. Pac. J. Trop. Biomed..

[3] World Health Organization. *WHO publishes list of bacteria for which new antibiotics are urgently needed*. (2017) Available at, https://www.who.int/news/item/27-02-2017-who-publishes-list-of-bacteria-for-which-new-antibiotics-are-urgently-needed (Date of access: 13/05/2022).

[4] Lai CC, Chen SY, Ko WC, Hsueh PR (2021). Increased antimicrobial resistance during the COVID-19 pandemic. Int. J. Antimicrob. Agents..

[5] Aurilio C (2021). Multidrug resistance prevalence in COVID area. Life (Basel).

[6] Pasupuleti M, Schmidtchen A, Malmsten M (2012). Antimicrobial peptides: key components of the innate immune system. Crit. Rev. Biotechnol..

[7] Li Y, Xiang Q, Zhang Q, Huang Y, Su Z (2012). Overview on the recent study of antimicrobial peptides: origins, functions, relative mechanisms and application. Peptides.

[8] Mookherjee N, Anderson MA, Haagsman HP, Davidson DJ (2020). Antimicrobial host defence peptides: functions and clinical potential. Nat. Rev. Drug Discov..

[9] Wang J (2018). Antimicrobial peptides: promising alternatives in the post feeding antibiotic era. Med. Res. Rev..

[10] Li J (2017). Membrane active antimicrobial peptides: translating mechanistic insights to design. Front. Neurosci..

[11] Zelezetsky I, Pag U, Sahl HG, Tossi A (2005). Tuning the biological properties of amphipathic alpha-helical antimicrobial peptides: rational use of minimal amino acid substitutions. Peptides.

[12] Bengtsson T (2020). Plantaricin NC8 exerts potent antimicrobial activity against *Staphylococcus* spp. and enhances the effects of antibiotics. Sci. Rep..

[13] Kumar P, Kizhakkedathu JN, Straus SK (2018). Antimicrobial peptides: diversity, mechanism of action and strategies to improve the activity and biocompatibility *in vivo*. Biomolecules.

[14] Kapil S, Sharma V (2021). d-Amino acids in antimicrobial peptides: a potential approach to treat and combat antimicrobial resistance. Can. J. Microbiol..

[15] Li Y (2019). Antimicrobial activity, membrane interaction and stability of the D-amino acid substituted analogs of antimicrobial peptide W3R6. J. Photochem. Photobiol. B Biol..

[16] Chen HL, Su PY, Shih C (2016). Improvement of *in vivo* antimicrobial activity of HBcARD peptides by D-arginine replacement. Appl. Microbiol. Biotechnol..

[17] Raheem N (2020). Insights into the mechanism of action of two analogues of aurein 2.2. Biochim. Biophys. Acta Biomembr..

[18] Klubthawee N, Adisakwattana P, Hanpithakpong W, Somsri S, Aunpad R (2020). A novel, rationally designed, hybrid antimicrobial peptide, inspired by cathelicidin and aurein, exhibits membrane-active mechanisms against *Pseudomonas aeruginosa*. Sci. Rep..

[19] Cockerill, F. R. Methods for dilution antimicrobial susceptibility tests for bacteria that grow aerobically; approved standard—ninth edition (ed. Wayne, P.) 16–19 (Clinical and Laboratory Standards Institute, 2012).

[20] Stark M, Liu LP, Deber CM (2002). Cationic hydrophobic peptides with antimicrobial activity. Antimicrob. Agents Chemother..

[21] Greco I (2020). Correlation between hemolytic activity, cytotoxicity and systemic *in vivo* toxicity of synthetic antimicrobial peptides. Sci. Rep..

[22] Zhang SK (2016). Design of an alpha-helical antimicrobial peptide with improved cell-selective and potent anti-biofilm activity. Sci. Rep..

[23] Xu W, Zhu X, Tan T, Li W, Shan A (2014). Design of embedded-hybrid antimicrobial peptides with enhanced cell selectivity and anti-biofilm activity. PLoS ONE.

[24] Liu Y, Xia X, Xu L, Wang Y (2013). Design of hybrid beta-hairpin peptides with enhanced cell specificity and potent anti-inflammatory activity. Biomaterials.

[25] Kwon JY (2019). Mechanism of action of antimicrobial peptide P5 truncations against *Pseudomonas aeruginosa* and *Staphylococcus aureus*. AMB Express.

[26] Harris, J. E. *Electron Microscopy in Biology: A Practical Approach* (ed. Harris, R.) 308 (Oxford University Press, 1991)

[27] Gabriel, B. L. *Biological Electron Microscopy* 264 (Van Nostrand Reinhold Company, 1982)

[28] Ma Z (2015). Characterization of cell selectivity, physiological stability and endotoxin neutralization capabilities of alpha-helix-based peptide amphiphiles. Biomaterials.

[29] Elliott AG (2020). An amphipathic peptide with antibiotic activity against multidrug-resistant Gram-negative bacteria. Nat. Commun..

[30] Muller PY, Milton MN (2012). The determination and interpretation of the therapeutic index in drug development. Nat. Rev. Drug Discov..

[31] Feng Z, Xu B (2016). Inspiration from the mirror: D-amino acid containing peptides in biomedical approaches. Biomol. Concepts.

[32] Fishbain J, Peleg AY (2010). Treatment of *Acinetobacter* infections. Clin. Infect. Dis..

[33] Yeaman MR, Gank KD, Bayer AS, Brass EP (2002). Synthetic peptides that exert antimicrobial activities in whole blood and blood-derived matrices. Antimicrob. Agents Chemother..

[34] International Organization for Standardization. *Biological evaluation of medical devices—part:5 tests for cytotoxicity (ISO EN 10993–5).* (2009) Available at https://nhiso.com/wp-content/uploads/2018/05/ISO-10993-5-2009.pdf (Date of access: 18/05/2022).

[35] He S (2020). Systematically studying the optimal amino acid distribution patterns of the amphiphilic structure by using the ultrashort amphiphiles. Front. Microbiol..

[36] Morris CJ (2012). Pegylation of antimicrobial peptides maintains the active peptide conformation, model membrane interactions, and antimicrobial activity while improving lung tissue biocompatibility following airway delivery. Antimicrob. Agents Chemother..

[37] Khan HA, Ahmad A, Mehboob R (2015). Nosocomial infections and their control strategies. Asian. Pac. J. Trop. Biomed..

[38] Lv Y (2014). Antimicrobial properties and membrane-active mechanism of a potential alpha-helical antimicrobial derived from cathelicidin PMAP-36. PLoS ONE.

[39] Wood SJ (2014). Modified cysteine-deleted tachyplesin (cdt) analogs as linear antimicrobial peptides: influence of chain length, positive charge, and hydrophobicity on antimicrobial and hemolytic activity. Int. J. Pept. Res. Ther..

[40] Chen Y (2007). Role of peptide hydrophobicity in the mechanism of action of alpha-helical antimicrobial peptides. Antimicrob. Agents Chemother..

[41] Huang Y, Huang J, Chen Y (2010). Alpha-helical cationic antimicrobial peptides: relationships of structure and function. Protein Cell.

[42] Svendsen JSM, Grant TM, Rennison D, Brimble MA, Svenson J (2019). Very short and stable lactoferricin-derived antimicrobial peptides: design principles and potential uses. Acc. Chem. Res..

[43] Mohamed MF, Brezden A, Mohammad H, Chmielewski J, Seleem MN (2017). A short D-enantiomeric antimicrobial peptide with potent immunomodulatory and antibiofilm activity against multidrug-resistant *Pseudomonas aeruginosa* and *Acinetobacter baumannii*. Sci. Rep..

[44] Vila-Farres X (2015). Sequence-activity relationship, and mechanism of action of mastoparan analogues against extended-drug resistant *Acinetobacter baumannii*. Eur. J. Med. Chem..

[45] Gorr SU, Flory CM, Schumacher RJ (2019). *In vivo* activity and low toxicity of the second-generation antimicrobial peptide DGL13K. PLoS ONE.

[46] Kerenga BK (2019). Salt-tolerant antifungal and antibacterial activities of the corn defensin ZmD32. Front. Microbiol..

[47] Sim JY (2019). A significantly enhanced antibacterial spectrum of D-enantiomeric lipopeptide bactenecin. Biochem. Biophys. Res. Commun..

[48] Krishnan M, Choi J, Jang A, Kim Y (2020). A novel peptide antibiotic, Pro10-1D, designed from insect defensin shows antibacterial and anti-inflammatory activities in sepsis models. Int. J. Mol. Sci..

[49] Huang Y (2014). Role of helicity of alpha-helical antimicrobial peptides to improve specificity. Protein Cell..

[50] Pandit G (2021). Effect of secondary structure and side chain length of hydrophobic amino acid residues on the antimicrobial activity and toxicity of 14-residue-long de novo AMPs. Chem. Med. Chem..

[51] Zhang QY (2021). Antimicrobial peptides: mechanism of action, activity and clinical potential. Mil. Med. Res..

[52] Ebenhan T, Gheysens O, Kruger HG, Zeevaart JR, Sathekge MM (2014). Antimicrobial peptides: their role as infection-selective tracers for molecular imaging. Biomed. Res. Int..

[53] Rishi P (2018). Efficacy of designer K11 antimicrobial peptide (a hybrid of melittin, cecropin A1 and magainin 2) against *Acinetobacter baumannii*-infected wounds. Pathog. Dis..

[54] Mwangi J (2019). The antimicrobial peptide ZY4 combats multidrug-resistant *Pseudomonas aeruginosa* and *Acinetobacter baumannii* infection. Proc. Natl. Acad. Sci. U. S. A..

