# Author Correction: Novel D-form of hybrid peptide (D-AP19) rapidly kills *Acinetobacter baumannii* while tolerating proteolytic enzymes

**DOI:** 10.1038/s41598-023-30354-z

**Published:** 2023-02-23

**Authors:** Phanvimon Jariyarattanarach, Natthaporn Klubthawee, Mathira Wongchai, Sittiruk Roytrakul, Ratchaneewan Aunpad

**Affiliations:** 1 Graduate Program in Biomedical Sciences, Faculty of Allied Health Sciences, Thammasat University, Khlong Luang, Pathum Thani 12120 Thailand; 2 Functional Ingredients and Food Innovation Research Group, National Center for Genetic Engineering and Biotechnology, National Science and Technology Development Agency, Khlong Luang, Pathum Thani 12120 Thailand

Correction to: *Scientific Reports* 10.1038/s41598-022-20236-1, published online 23 September 2022

The original version of this Article contained an error, where the COA number of the Ethics Committee of Thammasat University (COA No. 066/2562) was incorrectly given as ‘(COA No. 060/2564)’ in the Materials and Methods section.

As a result, in the Materials and methods, under the subheading ‘Hemolysis assay’,

“Experiments associated with human volunteers were carried out in accord with the ethical standards of and approved by the Ethics Committee of Thammasat University (COA No. 060/2564).”

now reads:

“Experiments associated with human volunteers were carried out in accord with the ethical standards of and approved by the Ethics Committee of Thammasat University (COA No. 066/2562).”

The original Article has been corrected.

