# Supplementary material for: Novel D-form of hybrid peptide (D-AP19) rapidly kills Acinetobacter baumannii while tolerating proteolytic enzymes
Source: Sci Rep. 2022 Sep 23;12:15852. doi: 10.1038/s41598-022-20236-1 (PMC9508196; doi:10.1038/s41598-022-20236-1)
Supplement: Supplementary file 1 — Supplementary Information. [file 41598_2022_20236_MOESM1_ESM.docx]

**Supplementary Information**

**Novel D-form of hybrid peptide (D-AP19) rapidly kills *Acinetobacter baumannii* while tolerating proteolytic enzymes**

Phanvimon Jariyarattanarach^1^, Natthaporn Klubthawee^1^, Mathira Wongchai^1^, Sittiruk Roytrakul^2^, Ratchaneewan Aunpad^1*^

^1^Graduate Program in Biomedical Sciences, Faculty of Allied Health Sciences, Thammasat University, Khlong Luang, Pathum Thani 12120, Thailand

^2^ Functional Ingredients and Food Innovation Research Group, National Center for Genetic Engineering and Biotechnology, National Science and Technology Development Agency, Khlong Luang, Pathum Thani 12120, Thailand

*Corresponding author

E-mail address: aratchan@tu.ac.th


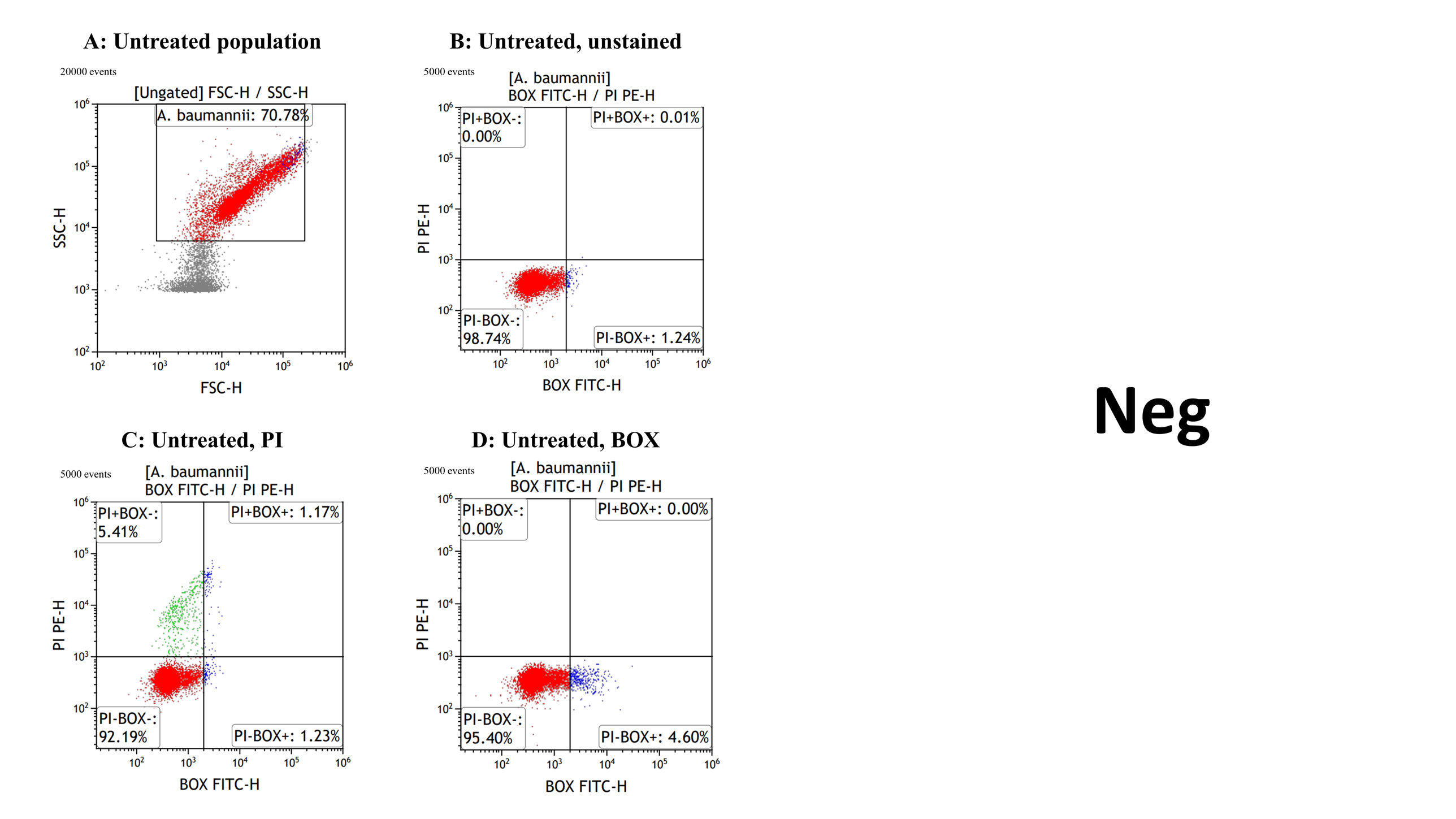


**Figure S1.** Flow cytometry analysis of untreated *A. baumannii* ATCC 19606 stained with PI or BOX, as a negative control of healthy cells. Five thousand events were recorded and plotted as dots in each quadrant (one dot represents one cell). Population of untreated bacterial cells (A), Untreated cells without PI or BOX staining (B), Untreated cells with PI staining (C), and Untreated cells with BOX staining (D). The percentage of cells located in each gate is exhibited at the corners of each box


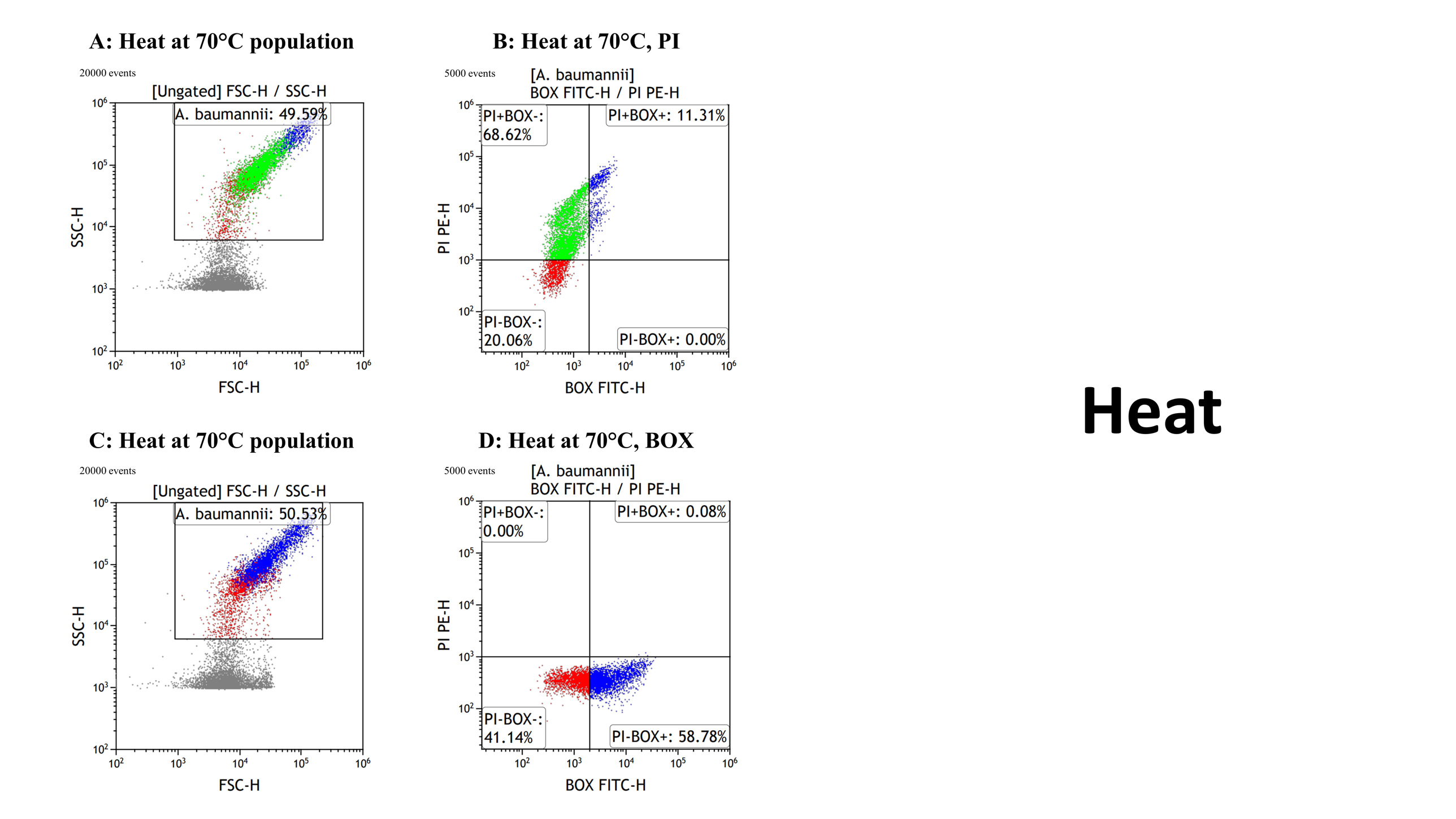


**Figure S2.** Flow cytometry analysis of *A. baumannii* ATCC 19606 stained with PI or BOX. Bacterial cells were heated at 70°C for 30 min to cause thermolysis of cells, for us as a positive control. Five thousand events were recorded and plotted as dots in each quadrant (one dot represents one cell). Population of heated bacterial cells with PI staining (A and B), and population of heated bacterial cells with BOX staining (C and D). The percentage of cells located in each gate is exhibited at the corners of each box.

**Table S1** Antibiotic susceptibility profile of eight clinical isolates of *A. baumannii*.

| ***A. baumannii*** **Isolates** | **Antibiotic susceptibility profile^a^** | | | | | | | |
| --- | --- | --- | --- | --- | --- | --- | --- | --- |
|  | **Caz** | **Tzp** | **Gen** | **Cip** | **Mem** | **Amp** | **Lvx** | **Cst** |
| A-8.2-1 | R | R | R | R | R | R | R | S |
| B-8.1-1 | S | R | I | S | S | R | S | S |
| B-8.1-2 | R | R | R | R | R | R | R | S |
| B-8.2-2 | R | R | R | R | R | R | R | S |
| C-8.1-2 | I | R | R | R | R | R | R | S |
| C-8.2-1 | R | R | R | R | R | R | R | S |
| D-10.1-1 | R | R | R | R | R | R | I | S |
| D-10.1-2 | I | R | I | R | R | R | R | S |

^a^Antibiotic susceptibility profile of all isolated strains was evaluated by disk diffusion test, using the standard protocol guide of the Clinical and Laboratory Standards Institute (CLSI), and interpreted as S: Susceptible to standard dosing regimen; I: Intermediate; R: Resistant (in accordance with the criteria of CLST breakpoints).

Note: Caz: Ceftazidime (third-generation cephalosporin class); Tzp: Piperacillin/tazobactam (penicillin class); Gen: Gentamicin (aminoglycoside class); Cip: Ciprofloxacin (fluoroquinolone class); Mem: Meropenem (carbapenem class); Amp: Ampicillin (penicillin class); Lvx: Levofloxacin (fluoroquinolone class); Cst: Colistin (polymyxin class).
